# Multimodal Artificial Intelligence‐Based Virtual Biopsy for Diagnosing Abdominal Lavage Cytology‐Positive Gastric Cancer

**DOI:** 10.1002/advs.202411490

**Published:** 2025-02-22

**Authors:** Ping'an Ding, Jiaxuan Yang, Honghai Guo, Jiaxiang Wu, Haotian Wu, Tongkun Li, Renjun Gu, Lilong Zhang, Jinchen He, Peigang Yang, Yuan Tian, Ning Meng, Xiaolong Li, Zhenjiang Guo, Lingjiao Meng, Qun Zhao

**Affiliations:** ^1^ The Third Department of Surgery the Fourth Hospital of Hebei Medical University Shijiazhuang 050011 China; ^2^ Hebei Key Laboratory of Precision Diagnosis and Comprehensive Treatment of Gastric Cancer Shijiazhuang 050011 China; ^3^ Big data analysis and mining application for precise diagnosis and treatment of gastric cancer Hebei Provincial Engineering Research Center Shijiazhuang 050011 China; ^4^ School of Chinese Medicine & School of Integrated Chinese and Western Medicine Nanjing University of Chinese Medicine Nanjing Jiangsu 210023 China; ^5^ Department of Gastroenterology and Hepatology Jinling Hospital Medical School of Nanjing University Nanjing Jiangsu 210002 China; ^6^ Department of General Surgery Renmin Hospital of Wuhan University Wuhan Hubei 430065 China; ^7^ Department of General Surgery Shijiazhuang People's Hospital Shijiazhuang Hebei 050050 China; ^8^ Department of General Surgery Baoding Central Hospital Baoding Hebei 071030 China; ^9^ General Surgery Department Hengshui People's Hospital Hengshui Hebei 053099 China; ^10^ Research Center and Tumor Research Institute the Fourth Hospital of Hebei Medical University Shijiazhuang 050011 China

**Keywords:** gastric cancer, multimodal artificial intelligence, peritoneal lavage cytology‐positive (CY1), radiomics, virtual biopsy

## Abstract

Gastric cancer with peritoneal dissemination remains a significant clinical challenge due to its poor prognosis and difficulty in early detection. This study introduces a multimodal artificial intelligence‐based risk stratification assessment (RSA) model, integrating radiomic and clinical data to predict peritoneal lavage cytology‐positive (GC‐CY1) in gastric cancer patients. The RSA model is trained and validated across retrospective, external, and prospective cohorts. In the training cohort, the RSA model achieved an area under the curve (AUC) of 0.866, outperforming traditional clinical and radiomic feature models. External validation cohorts confirmed its robustness, with AUC values of 0.883 and 0.823 for predicting peritoneal metastasis and recurrence, respectively. In a prospective validation involving 152 patients, the model maintained superior predictive performance (AUC = 0.835). The RSA model also demonstrated significant clinical benefits by effectively identifying high‐risk patients likely to benefit from specific treatments, such as paclitaxel‐based conversion therapy. These findings suggest that the RSA model offers a reliable, non‐invasive diagnostic tool for gastric cancer, capable of improving early detection and treatment outcomes. Further prospective studies are warranted to explore its full clinical potential.

## Graphical abstract

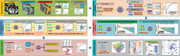

## 1 Introduction

Gastric cancer remains a significant global health burden, ranking as one of the leading causes of cancer‐related mortality worldwide.^[^ ^1^ ^]^ Despite advances in treatment, the prognosis for patients with advanced gastric cancer, particularly those with peritoneal dissemination, remains poor.^[^ ^2^, ^3^ ^]^ Peritoneal lavage cytology is an established diagnostic method used to detect free cancer cells within the peritoneal cavity, serving as a critical indicator of occult peritoneal metastasis.^[^ ^4^ ^]^ Patients with peritoneal lavage cytology‐positive (GC‐CY1) status often face a high risk of recurrence and reduced survival rates, even in the absence of visible peritoneal disease, necessitating more aggressive treatment strategies.^[^ ^5^, ^6^ ^]^ Accurate detection of CY1‐positive status is therefore crucial for optimizing therapeutic decisions and improving patient outcomes.

Currently, the standard diagnostic approaches for detecting peritoneal involvement in gastric cancer include computed tomography (CT) and laparoscopic exploration.^[^ ^7^, ^8^ ^]^ CT imaging is widely utilized for the preoperative staging of gastric cancer, providing valuable information on the presence of distant metastases and the extent of the primary tumor.^[^ ^9^ ^]^ However, CT has limited sensitivity for detecting early or microscopic peritoneal metastasis, often failing to identify small peritoneal nodules or free cancer cells in the peritoneal cavity.^[^ ^10^ ^]^ As a result, many patients with peritoneal involvement remain undetected until the disease has significantly progressed, thereby limiting treatment options and negatively impacting prognosis. Many new diagnostic methods are now emerging that may provide new tools for identifying cancer patients. For example, recent advancements in nanotechnology have introduced innovative diagnostic strategies, such as tumor‐targeting nanorobots, which can precisely recognize tumor cells and offer potential applications in early cancer detection.^[^ ^11^, ^12^ ^]^ However, these approaches are still in the early stages of exploration and require further validation before they can be truly applied in clinical practice.

Laparoscopic exploration, another common diagnostic tool, offers a more direct method of evaluating the peritoneal cavity and detecting peritoneal metastases.^[^ ^13^, ^14^ ^]^ While it provides higher sensitivity compared to CT, it is an invasive procedure that carries risks associated with surgery, such as infection, bleeding, and anesthesia‐related complications.^[^ ^15^ ^]^ Moreover, laparoscopy is limited by its dependence on the operator's expertise and the potential for sampling error, as small or isolated peritoneal metastases may be missed.^[^ ^16^ ^]^ Due to these limitations, there is an urgent need for more advanced, non‐invasive diagnostic methods that can reliably detect GC‐CY1 at earlier stages, enabling timely and targeted therapeutic interventions.

The advent of artificial intelligence (AI) and machine learning (ML) has introduced new possibilities for enhancing cancer diagnostics through the development of virtual biopsy technologies.^[^ ^17^, ^18^ ^]^ These technologies utilize AI algorithms to analyze multimodal data (including imaging, cytology, genomics, and histopathology) without the need for invasive tissue sampling. By integrating multiple data sources, multimodal AI‐based approaches can provide a comprehensive and precise assessment of cancer status, potentially overcoming the limitations of traditional diagnostic methods.^[^ ^19^, ^20^ ^]^ In particular, virtual biopsy technology holds promise for improving the detection of GC‐CY1, offering a non‐invasive, accurate, and rapid alternative to current diagnostic standards.

This study aims to evaluate the use of multimodal AI‐based virtual biopsy technology for diagnosing GC‐CY1. We propose a novel diagnostic approach that integrates advanced AI techniques with diverse data types to enhance the detection of peritoneal metastasis. Our research explores the potential of this technology to improve diagnostic accuracy and sensitivity, reduce the need for invasive procedures, and ultimately contribute to better patient management and outcomes.

## 2 Methods and Materials

### 2.1 Study Cohort

**Figure 1:**
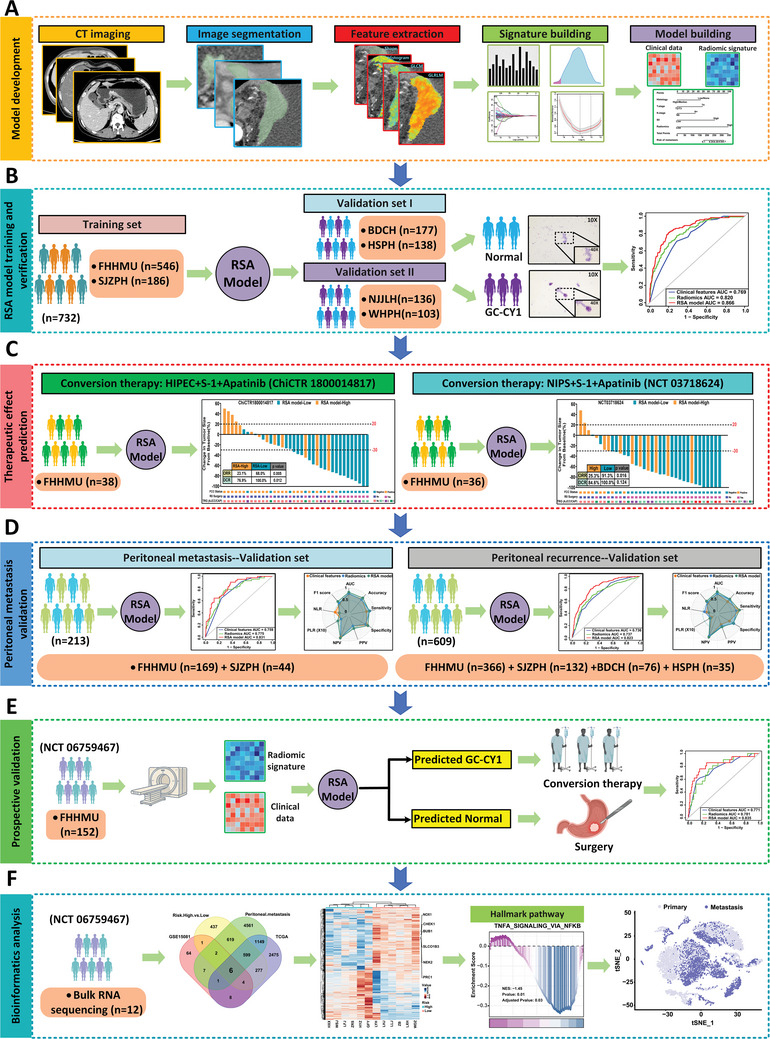
Study design flow chart. This study involved 2334 eligible patients with LAGC from six medical centers in China, all of whom underwent abdominal CT imaging before treatment. A,B) Radiology workflow: The process included manual segmentation after image acquisition, feature extraction, and the establishment of radiomic features, followed by the development and validation of the RSA model to predict CY1‐positivity. The model's performance was then evaluated. C) Prediction of conversion therapy efficacy: A retrospective analysis of two cohorts of GC‐CY1 patients who previously underwent conversion therapy was conducted to explore the relationship between the RSA model and treatment efficacy. D) Prediction of peritoneal metastasis and recurrence: An extended analysis of the study cohort was carried out to predict peritoneal metastasis in newly diagnosed gastric cancer patients and peritoneal recurrence after radical surgery using the RSA model. E) Prospective validation: A total of 152 patients with LAGC were prospectively enrolled to validate the RSA model's predictive performance for CY1‐positivity (NCT 06759467). F) Bioinformatics workflow: RNA sequencing was performed on 12 tissue samples from the prospective cohort. Bioinformatics analysis was conducted to investigate the biological characteristics and immune infiltration features of GC‐CY1 patients, providing insights into intratumor heterogeneity.

This study analyzed seven retrospective datasets and one prospective dataset (NCT 06759467) from six medical centers in China, and details of these are provided in Table S1 (Supporting Information). The retrospective study of GC‐CY1 data were collected between 2013 and 2019, comprising a total of 1286 patients with locally advanced gastric cancer (LAGC) (**Figure** **1**). The training set consisted of 732 patients, including 546 from the Fourth Hospital of Hebei Medical University (FHHMU) and 186 from Shijiazhuang People's Hospital (SJZPH). The first external validation cohort included 315 patients from two additional medical centers in Hebei Province [Baoding Central Hospital (BDCH) and Hengshui People's Hospital (HSPH)]. To further assess the model's generalizability and robustness, a second external validation cohort of 239 patients was selected from two other medical centers [Wuhan University People's Hospital (WHPH) and Nanjing Jinling Hospital (NJJLH)] in China. The prospective dataset, part of the clinical trial NCT 06759467, was collected from January to June 2024 and included 152 patients with LAGC.

To further assess the broad applicability and clinical utility of the model, we retrospectively analyzed cohorts from two clinical trials on conversion therapy for GC‐CY1 patients registered at the FHHMU [ChiCTR 1 800 014 817 (*n* = 38) and NCT 0 371 8624 (*n* = 36)] to evaluate the model's performance in predicting treatment efficacy. Furthermore, we enrolled 213 patients with LAGC from FHHMU and SJZPH who underwent laparoscopic exploration to determine whether the model could be extended to predict peritoneal metastasis. Simultaneously, we identified 609 gastric cancer patients who underwent radical surgery at four medical centers in Hebei Province, using follow‐up data to establish a postoperative peritoneal recurrence validation cohort. This cohort was used to assess the model's ability to predict peritoneal recurrence after radical surgery.

To further validate the clinical applicability of the model, we initiated a prospective clinical study (NCT 06759467). From January to June 2024, we prospectively recruited 152 patients with LAGC at the FHHMU, following strict inclusion and exclusion criteria. To evaluate the practical application of the model, we also recruited 12 radiologists from 4 centers nationwide: 2 experts (with >10 years of experience), 4 senior radiologists (with >5 years of experience), and 6 novice radiologists (with 1–2 years of experience). None of the radiologists were involved in data collection or preprocessing and were blinded to patient information, CT reports, pathological results, and clinical diagnoses. An independent researcher (PAD) randomly assigned the radiologists to either a group using AI‐assisted diagnosis first or a group not using AI‐assisted diagnosis, in a 1:1 ratio, based on their professional experience (PAD was blinded to the radiologists' identities). After a 2‐week washout period, the groups were switched to further assess the clinical effectiveness and practical application of the model.

Gastric cancer patients with baseline RNA sequencing data were selected from the Cancer Genome Atlas (TCGA) databases for genomic immune infiltration analysis. In a prospective dataset (NCT 06759467), RNA sequencing was performed on 12 fresh tumor samples for exploratory purposes. Functional enrichment analysis was conducted to investigate the biological roles of the identified features. Additionally, using data from the TCGA and gene expression omnibus (GEO) databases, Cell‐type Identification by Estimating Relative Subsets of RNA Transcripts (CIBERSORT) and microenvironment cell populations‐counter (MCPcounter) were employed to infer cell types, states, and multicellular communities (referred to as cancer ecosystems) to elucidate immune infiltration.

All patients underwent a pretreatment abdominal CT scan and were restaged according to the 8th edition of the American Joint Committee on Cancer (AJCC) staging system. Figure S1 (Supporting Information) provides detailed information on the dataset, as well as the inclusion and exclusion criteria and treatment regimen, while Supporting Materials outlines the CT scanning protocol. The study was approved by the Institutional Review Board of the FHHMU. Written informed consent was obtained from all prospectively enrolled participants for mRNA transcriptomic sequencing. For the retrospective component of this observational study, written informed consent was not required.

### 2.2 Image Acquisition and Segmentation

In this study, patients underwent contrast‐enhanced abdominal CT scans before therapy, specifically obtaining portal venous phase images through a picture archiving and communication system. Segmentation was performed using 3D Slicer software (version 5.6.1; https://www.slicer.org), and the slice with the largest tumor area was selected as the region of interest. Two experienced radiation oncologists delineated the regions of interest, and a senior radiation oncologist with 15 years of experience evaluated each delineation for intra‐ and inter‐reader agreement.

### 2.3 Radiomic Signature Engineering

Feature engineering was performed using 3D Slicer software (version 5.6.1; https://www.slicer.org). To standardize images from different CT instruments, the CT images were resampled to a voxel size of 1 × 1 × 1 mm with a bin width of 25 for voxel intensities. A total of 1130 radiomic features were extracted from the region of interest on each patient's CT scan (Supporting Materials for further details).

### 2.4 Radiomics Features Selection and Radiomics Model Construction

Before feature selection, we normalized the features to account for differences in scale. Due to the high redundancy in the original feature set, a univariate analysis was initially performed to remove irrelevant features. The least absolute shrinkage and selection operator (LASSO) algorithm was then used for further feature reduction. The selection of the regularization parameter λ was performed using tenfold cross‐validation with deviance minimization as the performance metric. Specifically, we used the one‐standard error criterion to identify the λ value that achieves a balance between model complexity and performance. Finally, multivariate logistic regression was applied to fit the optimal feature set and generate the radiomic signature.

### 2.5 Clinical Model Construction

To select clinical variables for inclusion in the model, we performed multivariate logistic regression analysis. All collected patient characteristics, including sex, age, tumor location, maximum tumor diameter, pathological type, T stage, N stage, Eastern Cooperative Oncology Group (ECOG) score, prognostic nutritional index (PNI), neutrophil to lymphocyte ratio (NLR), systemic immune‐inflammation index (SII), and platelet to lymphpcyte ratio (PLR), were included in the initial analysis (Supporting Materials for further details). Features with *p* < 0.05 in the multivariate logistic regression were considered statistically significant and subsequently used for model construction.

### 2.6 Nomogram Construction

A nomogram was constructed based on the radiomic signature. To compare the radiomic signature with clinical predictors, two additional nomograms were developed: one using clinical factors alone (clinical model) and another combining the radiomic signature with selected clinical factors (comprehensive model). The predictive abilities of these models for GC‐CY1 were evaluated using receiver operating characteristic (ROC) curve analysis, and the area under the curve (AUC) was used to assess the performance of radiomic imaging biomarkers across different cohorts. To stratify patients into high‐risk and low‐risk groups, we performed ROC curve analysis. The maximum Youden index was used as the cutoff value to define the optimal threshold for risk group classification. The Youden index is calculated as sensitivity + specificity – 1, providing the point on the ROC curve that achieves the best trade‐off between sensitivity and specificity.

### 2.7 Statistical Analysis

All statistical analyses were performed using SPSS version 28.0 (IBM) and R version 4.3.3 (http://www.r‐project.org). Missing data were addressed using multiple imputation by chained equations (MICE) through the mice package in R. Continuous variables were analyzed using an unpaired two‐tailed *t*‐test and the Mann–Whitney U test, while categorical variables were evaluated using the *X* ^2^test and Fisher's exact test. Univariate and multivariate Cox regression analyses were conducted to assess the prognostic ability of the variables for survival. ROC curves were used to evaluate the predictive performance of the models, and the AUC values were calculated with 95% confidence intervals (CI) using DeLong's method. Missing data were handled using Central Tendency Imputation techniques to ensure the robustness of the analysis and reduce potential bias. Detailed algorithms for the statistical methods are provided in Supporting Materials. A two‐sided P‐value of less than 0.05 was considered statistically significant.

### 2.8 Ethics Approval and Consent to Participate

The study protocol was approved by the Ethics Committee of the FHHMU (approval number: 2024KY199). For the prospective component of the study, written informed consent was obtained from all participants prior to their inclusion. For the retrospective component, the requirement for written informed consent was waived by the Ethics Committee due to the use of de‐identified patient data, minimal risk to participants, and the retrospective nature of the analysis. The study was conducted in accordance with the ethical principles of the Declaration of Helsinki, and all authors adhered to applicable ethical standards to ensure research integrity, including the avoidance of duplication, fraud, and plagiarism.

### 2.9 Ethical Statement

All authors certify that they comply with the ethical guidelines for authorship.

## 3 Results

### 3.1 Patient Characteristics

A total of 1286 eligible patients were included in this study to develop and validate the GC‐CY1 prediction model. The cohort consisted of 984 (76.5%) males and 302 (23.5%) females, with a mean age of 58 (IQR: 51.0–65.0) years. All patients underwent laparoscopic exploration and peritoneal lavage fluid testing, with CY1‐positivity observed in 121 (16.5%), 52 (16.5%), and 35 (14.6%) patients across the three cohorts. The clinical characteristics of the 1286 patients in the training set and the two external validation sets are summarized in **Table** **1**. The distribution of characteristics among the three data sets was similar (all *p* > 0.05).

**Table 1:** Patient characteristics in the training and validation cohorts.

Characteristic	Overall	Training cohort	Validation cohort I	Validation cohort II
	*n* = 1286	*n* = 732	*n* = 315	*n* = 239
**Gender**				
Male	984 (76.5%)	559 (76.4%)	241 (76.5%)	184 (77.0%)
Female	302 (23.5%)	173 (23.6%)	74 (23.5%)	55 (23.0%)
**Age (IQR, years)**	58.0 (51.0–65.0)	57.0 (50.0–64.0)	58.0 (50.0–64.0)	61.0 (53.0–68.0)
≤65	872 (67.8%)	480 (65.6%)	230 (73.0%)	162 (67.8%)
>65	407 (32.2%)	252 (34.4%)	85 (27.0%)	77 (32.2%)
**ECOG PS**				
0–1	1128 (87.7%)	655 (89.5%)	269 (85.4%)	204 (85.4%)
2	158 (12.3%)	77 (10.5%)	46 (14.6%)	35 (14.6%)
**T stage**				
T2/T3	365 (28.4%)	182 (24.9%)	107 (34.0%)	76 (31.8%)
T4	921 (71.6%)	550 (75.1%)	208 (66.0%)	163 (68.2%)
**N stage**				
N0	372 (28.9%)	193 (26.4%)	100 (31.7%)	79 (33.1%)
N+	914 (71.1%)	539 (73.6%)	215 (68.3%)	160 (66.9%)
**Primary site**				
Up 1/3	451 (35.0%)	260 (35.5%)	100 (31.7%)	91 (38.1%)
Middle 1/3	213 (16.6%)	120 (16.4%)	55 (17.5%)	38 (15.9%)
Lower 1/3	622 (48.4%)	352 (48.1%)	160 (50.8%)	110 (46.0%)
**Borrmann type**				
I/II	411 (32.0%)	257 (35.1%)	89 (28.3%)	65 (27.2%)
III/IV	875 (68.0%)	475 (64.9%)	226 (71.7%)	174 (72.8%)
**Tumor size (IQR, cm)**	4.6 (3.8–6.5)	4.8 (4.1–6.8)	4.4 (3.3–5.8)	5.1 (4.1–7.1)
≤5	618 (48.1%)	356 (48.6%)	146 (46.3%)	116 (48.5%)
>5	668 (51.9%)	376 (51.4%)	169 (53.7%)	123 (51.5%)
**Histology**				
None/Low	992 (77.1%)	562 (76.8%)	259 (82.2%)	171 (71.5%)
High/Median	294 (22.9%)	170 (23.2%)	56 (17.8%)	68 (28.5%)
SII^*^				
Low	561 (43.6%)	346 (47.3%)	113 (35.9%)	102 (42.7%)
High	725 (56.4%)	386 (52.7%)	202 (64.1%)	137 (57.3%)
PNI^*^				
Low	222 (17.3%)	109 (14.9%)	83 (26.3%)	30 (12.6%)
High	1064 (82.7%)	623 (85.1%)	232 (76.7%)	209 (87.4%)
NLR^*^				
Low	817 (63.5%)	464 (63.4%)	196 (62.2%)	157 (65.7%)
High	469 (36.5%)	268 (36.6%)	119 (37.8%)	82 (34.3%)
PLR^*^				
Low	640 (49.8%)	370 (50.5%)	146 (46.3%)	124 (51.9%)
High	646 (50.2%)	362 (49.5%)	169 (53.7%)	115 (48.1%)

ECOG PS = Eastern cooperative oncology group performance status; SII = Systemic immune‐inflammation index; PNI = Prognostic nutritional index; NLR = Neutrophil to lymphocyte ratio; PLR = Platelet to lymphpcyte ratio. * Using the median expression as the threshold, the cells were divided into high group and low group.

### 3.2 Prediction Model Construction and Internal Validation

During the feature selection process, 936 radiomic features with intra‐ and inter‐observer intraclass correlation coefficients greater than 0.75 were included. Univariate analysis identified 732 statistically significant radiomic features, from which 10 were selected using the LASSO method (Figure S2, Supporting Information). These 10 features were subsequently used in a multivariate logistic regression analysis as independent prognostic predictors of GC‐CY1 (Table S2, Supporting Information). A machine learning nomogram was then developed using these 10 features, weighted by their respective regression coefficients (Figure S3, Supporting Information).

**Figure 2:**
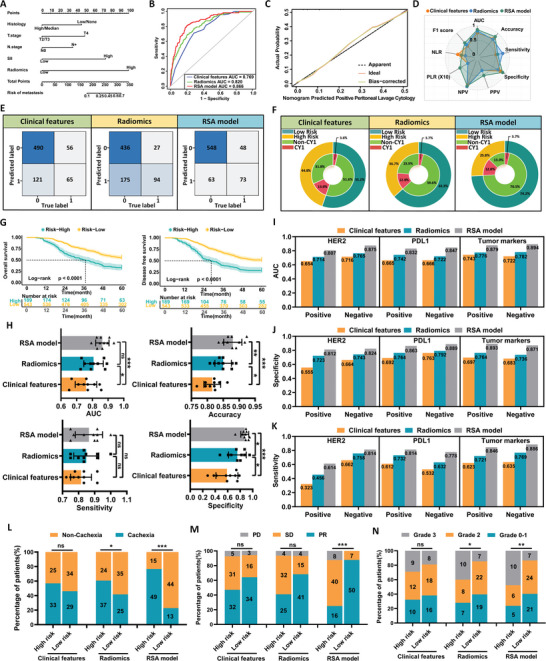
Training and Internal Validation of the RSA Model for Predicting GC‐CY1. A) Development of a nomogram incorporating radiomic features and clinical characteristics to predict GC‐CY1. B) ROC curves of various prediction models in the training set. C) Calibration curves of the RSA model in the training set. D) Radar plots illustrating performance indicators for different prediction models in the training set. E) Confusion matrices for the different prediction models in the training set. F) Double‐layer concentric circle plots demonstrating the clinical benefits of various prediction models in the training set. G) Log‐rank test survival curves for patients in the training set, stratified into low‐ and high‐risk groups based on the Youden index threshold derived from the nomogram. H) Box plots of AUC, sensitivity, specificity, and accuracy analyses for different prediction models after tenfold cross‐validation. I) Comparison of AUC values among different prediction models in a stratified analysis based on peripheral blood tumor markers and HER2 and PDL1 expression in biopsy tissues. J) Specificity comparison among different prediction models in a stratified analysis based on the expression status of HER2 and PDL1 in peripheral blood tumor markers and biopsy tissues. K) Sensitivity comparison among different prediction models in a stratified analysis based on the expression status of HER2 and PDL1 in peripheral blood tumor markers and biopsy tissues. L) Relationship between high‐ and low‐risk groupings of different prediction models and cachexia in GC‐CY1 patients. M) Relationship between high‐ and low‐risk groupings of different prediction models and conversion therapy efficacy in GC‐CY1 patients. N) Relationship between high‐ and low‐risk groupings of different prediction models and postoperative pathological regression grade after conversion therapy in GC‐CY1 patients.

Further univariate and multivariate analyses identified pathological type, infiltration depth (T stage), lymph node metastasis (N stage), and the peripheral blood SII as independent factors influencing GC‐CY1 (Table S3, Supporting Information). A comprehensive nomogram was constructed to assess whether combining radiomic features with clinical variables provided complementary predictive value over individual features (**Figure** **2**A). The combination of radiomic features and clinical variables resulted in the development of the Risk Stratification Assessment (RSA) model, which demonstrated strong predictive ability for GC‐CY1, with an AUC of 0.866 (95% CI: 0.829–0.920; Figure 2B,D,E). A detailed comparison of different models is provided in Table S4 (Supporting Information). The DeLong test indicated that the RSA model's AUC was significantly higher than that of the clinical model in the training set (0.866 versus 0.769; *p* = 0.001). The model's calibration curve further highlighted its predictive accuracy (Figure 2C). Based on the Youden index, patients were classified into low‐risk and high‐risk groups. The two‐layer concentric circle plot revealed that the RSA model identified more low‐risk GC‐CY1 patients than the clinical model (74.2% versus 55.2%) (Figure 2F). Additionally, the clinical impact curve confirmed that the RSA model had the greatest clinical benefit (Figure S4A–C, Supporting Information).

Follow‐up data showed that the 5‐year overall survival (OS) and disease‐free survival (DFS) rates were significantly lower in high‐risk patients compared to low‐risk patients (33.3% versus 55.6% and 29.1% versus 51.9%, respectively; *p* < 0.001) (Figure 2G). Cox proportional hazard regression analysis further confirmed the above results. Compared with high‐risk patients, the hazard ratio (HR) of OS in low‐risk patients was 0.488 (95% CI: 0.394‐0.606, *p* < 0.001), and the HR of DFS was 0.472 (95% CI: 0.383‐0.581, *p* < 0.001) (Table S12, Supporting Information). The results showed that the risk of recurrence and death in the high‐risk group was significantly higher than that in the low‐risk group. The AUC box plot, generated after tenfold cross‐validation demonstrated that the RSA model had superior discrimination ability, sensitivity, and specificity compared to the clinical and radiomics model (Figure 2H). Stratified analysis based on different peripheral blood tumor markers, HER2 and PDL1 expression in tissue biopsies also showed that the RSA model outperformed both the clinical and radiomics model in clinical prediction (Figure 2I–K). Additionally, the study systematically analyzed treatment strategies for all GC‐CY1 patients in the training set. Notably, the RSA model's risk stratification was more effective in identifying patients with cachexia (Figure 2L) and those likely to benefit from paclitaxel‐based conversion therapy (Figure 2M,N) than the clinical and radiomics model. A detailed paclitaxel‐based conversion therapy protocol is provided in Supporting Materials.

### 3.3 Multicenter External Validation of the Prediction Model

**Figure 3:**
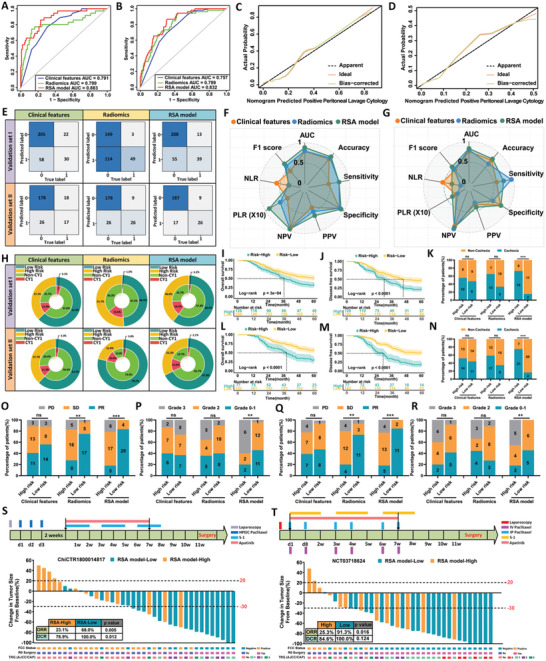
External validation of the RSA model for predicting GC‐CY1. A,B) ROC curves for different prediction models in two external validation sets. C,D) Calibration curves of the RSA model in two external validation sets. E) Confusion matrices for different prediction models in the two external validation sets. F,G) Radar plots displaying performance indicators for different prediction models in the two external validation sets. H) Double‐layer concentric circle plots illustrating the clinical advantages of various prediction models in the two external validation sets. I,J) Log‐rank test survival curves for patients in external validation set I, stratified into low‐ and high‐risk groups based on the Youden index threshold derived from the nomogram. K) Comparison of the relationship between high‐ and low‐risk groups from different prediction models in external validation set I and cachexia in GC‐CY1 patients. L,M) Log‐rank test survival curves for patients in external validation set II, stratified into low‐ and high‐risk groups according to the Youden index threshold derived from the nomogram. N) Comparison of the relationship between high‐ and low‐risk groups from different prediction models in external validation set II and cachexia in GC‐CY1 patients. O) Comparison of the relationship between high‐ and low‐risk groups from different prediction models in external validation set I and conversion therapy efficacy in GC‐CY1 patients. P) Comparison of the relationship between high‐ and low‐risk groups from different prediction models in external validation set I and postoperative pathological regression grade after conversion therapy in GC‐CY1 patients. Q) Analysis of the relationship between high‐ and low‐risk groups from different prediction models in external validation set II and conversion therapy efficacy in GC‐CY1 patients. R) Analysis of the relationship between high‐ and low‐risk groups from different prediction models in external validation set II and postoperative pathological regression grade after conversion therapy in GC‐CY1 patients. S,T) Retrospective analysis of two prospective cohorts of GC‐CY1 patients undergoing conversion therapy (ChiCTR1800014817, NCT03718624) to investigate the relationship between the RSA model and treatment efficacy.

To further validate the model's generalizability, we employed a two‐step external validation approach. First, 315 gastric cancer patients from two medical centers in Hebei Province (BDCH and HSPH) were selected as the first external validation cohort (validation set I). To account for regional differences, we also included 239 patients from two other medical centers (WHPH and NJJLH) in China as a second external validation cohort (validation set II). The same statistical parameters from the training cohort were applied to both validation cohorts, ensuring consistency across datasets. In validation set I, the RSA model demonstrated superior predictive ability compared to both the clinical feature model and the radiomic feature model (AUC = 0.883, 95% CI: 0.824–0.943; **Figure** **3**A, E upper, F; Table S4, Supporting Information). Calibration curve analysis further confirmed the enhanced predictive accuracy (Figure 3C). Similar results were observed in the second external validation set (Figure 3B, D, E lower, G; Table S4, Supporting Information).

As shown in Figure 3H, a double‐layer concentric circle plot revealed that in both validation sets, the RSA model identified more GC‐CY1 patients in the high‐risk group compared to the clinical feature model (validation set I: 14.3% versus 11.4%; validation set II: 12.6% versus 11.3%). In the low‐risk group, the RSA model also more accurately identified non‐GC‐CY1 patients (validation set I: 2.2% versus 5.1%; validation set II: 2.1% versus 3.3%). Additionally, the clinical impact curve (CIC) showed that the RSA model provided the best performance across both external validation sets (Figure S4D–I, Supporting Information). In both validation cohorts, the RSA model effectively stratified gastir cancer patients, with low‐risk patients showing significantly higher 5‐year OS (51.3% versus 31.7% in set I; 56.8% versus 29.9% in set II) and DFS (46.6% versus 21.4% in set I; 51.2% versus 18.2% in set II) compared to high‐risk patients (all *p* < 0.001) (Figure 3I,J,L,M; Table S12, Supporting Information).

We also assessed the effectiveness of conversion therapy and the occurrence of cachexia in GC‐CY1 patients across the two external validation sets. Consistent with the training set results, the RSA model was more effective than the clinical and radiomic feature models in identifying patients with cachexia (Figure 3K,N) and those likely to benefit from paclitaxel‐based conversion therapy (Figure 3O–R). To further verify the RSA model's effectiveness in predicting conversion therapy outcomes in GC‐CY1 patients, we retrospectively analyzed data from two prospective clinical studies conducted at the FHHMU [ChiCTR 1 800 014 817 (*n* = 38) and NCT 0 371 8624 (*n* = 36)]. The clinical characteristics of these patients are detailed in Table S5 (Supporting Information). Based on the RSA model's high‐ and low‐risk stratification, both studies revealed that the objective response rate (ORR) and disease control rate (DCR) were significantly worse for high‐risk patients compared to low‐risk patients after receiving paclitaxel‐based conversion therapy (ORR: ChiCTR 1 800 014 817: 23.1% versus 68.0%, *p* = 0.005; NCT 0 371 8624: 25.3% versus 91.3%, *p* = 0.016; DCR: ChiCTR 1 800 014 817: 76.9% versus 100.0%, *p* = 0.012; NCT 0 371 8624: 84.6% versus 100.0%, *p* = 0.124; Figure 3S,T).

### 3.4 Extended Validation of the Prediction Model for Peritoneal Metastasis and Recurrence

**Figure 4:**
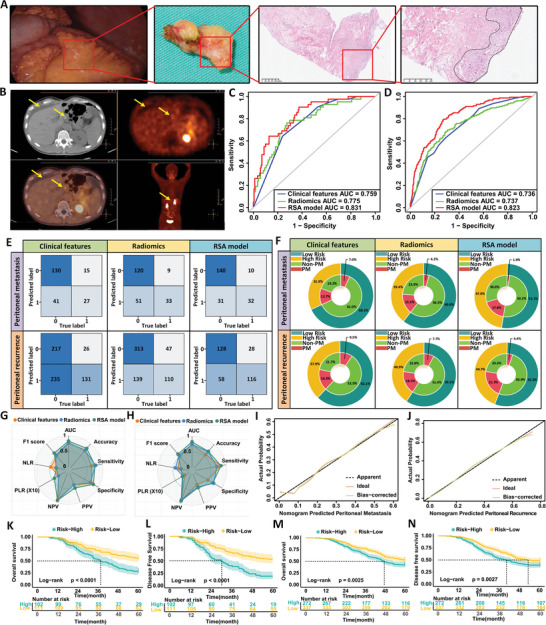
Extended validation of the RSA model for predicting peritoneal metastasis and recurrence. A) Standard peritoneal biopsy procedure and HE staining results used for diagnosing peritoneal metastasis in newly diagnosed gastric cancer patients undergoing laparoscopic exploration. B) Typical PET‐CT images showing peritoneal recurrence during follow‐up of patients after radical surgery for gastric cancer. C) ROC curves for different prediction models in the peritoneal metastasis validation set. D) ROC curves for different prediction models in the peritoneal recurrence validation set. E) Confusion matrices for various prediction models in both the peritoneal metastasis and peritoneal recurrence validation sets. F) Double‐layer concentric circle plots illustrating the clinical advantages of different prediction models in the peritoneal metastasis and recurrence validation sets. G,H) Radar plots showing the performance indicators of different prediction models in the peritoneal metastasis and recurrence validation sets. I) Calibration curve of the RSA model in the peritoneal metastasis validation set. J) Calibration curve of the RSA model in the peritoneal recurrence validation set. K,L) Log‐rank test survival curves of patients with peritoneal metastasis in the validation set, categorized into low‐risk and high‐risk groups based on the Youden index thresholds derived from the nomogram. M,N) Log‐rank test survival curves of patients with peritoneal recurrence in the validation set, categorized into low‐risk and high‐risk groups according to the Youden index thresholds derived from the nomogram.

Based on the “seed and soil theory,” if free cancer cells in the abdominal cavity are not promptly treated, they may lead to visible peritoneal metastasis and subsequently worsen the patient's prognosis. To evaluate whether the RSA model can be extended to predict peritoneal metastasis in newly diagnosed patients and peritoneal recurrence after radical surgery, we retrospectively selected 213 patients from the FHHMU and SJZPH as the peritoneal metastasis validation cohort. The clinical characteristics of these patients are presented in Table S6 (Supporting Information). All patients underwent laparoscopic exploration and peritoneal biopsy to diagnose peritoneal metastasis (**Figure** **4**A). Additionally, we identified 609 gastric cancer patients who underwent radical surgery at four medical centers in Hebei Province (FHHMU, SJZPH, BDCH and HSPH) and were subsequently found to have peritoneal recurrence through postoperative follow‐up. These patients formed the postoperative peritoneal recurrence validation cohort, with detailed information provided in Table S7 (Supporting Information). For these patients, whole‐body PET‐CT scans served as the gold standard for diagnosing peritoneal recurrence (Figure 4B).

The RSA model demonstrated a strong ability to predict peritoneal metastasis, with an AUC of 0.831 (95% CI: 0.767–0.895), outperforming the clinical and radiomics model (Figure 4C,E,G; Table S8, Supporting Information). Calibration curve analysis further confirmed its superior predictive accuracy (Figure 4I). The double‐layer concentric circle plot showed that the RSA model identified more patients with peritoneal metastasis in the high‐risk group compared to the clinical feature model (17.8% versus 12.7%) and more accurately identified non‐metastasis cases in the low‐risk group (1.9% versus 7.0%, Figure 4F upper). Clinical impact curve analysis further supported the RSA model's greater clinical benefit compared to the other models (Figure S5A–C, Supporting Information).

Similar results were observed in the external validation set for predicting peritoneal recurrence after radical surgery, where the RSA model also showed strong predictive ability (AUC = 0.823, 95% CI: 0.787–0.859; Figure 4D,E,H,J; Table S8, Supporting Information). Additionally, the RSA model enabled more accurate stratification of postoperative peritoneal recurrence risk (Figure 4F lower; Figure S5D–F, Supporting Information).

Survival follow‐up of all LAGC patients in the two validation sets revealed that low‐risk patients, as stratified by the RSA model, had significantly better 5‐year OS and DFS rates compared to high‐risk patients. Low‐risk patients showed significantly better outcomes in both validation cohorts, with higher 5‐year OS and DFS rates in the peritoneal metastasis cohort (55.9% versus 28.4% and 53.2% versus 18.6%, respectively) and the postoperative peritoneal recurrence cohort (53.1% versus 42.6% and 48.7% versus 39.3%, respectively) compared to high‐risk patients (all *p* < 0.001) (Figure 4K–N, Table S12, Supporting Information).

### 3.5 Prospective Validation of the Prediction Model

**Figure 5:**
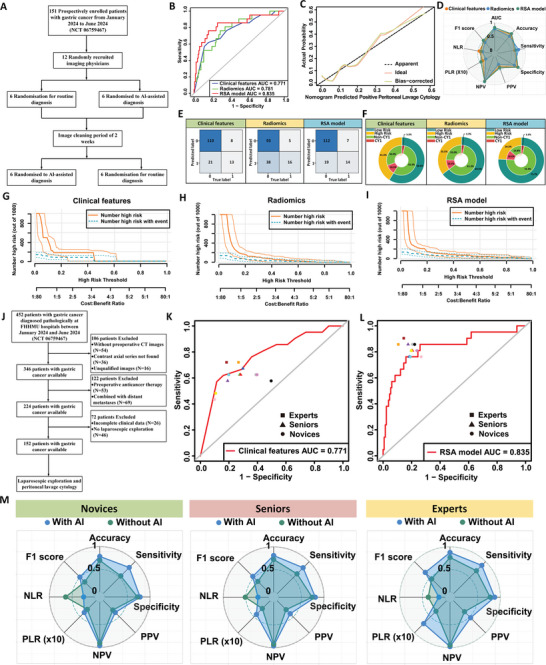
Prospective Validation of the RSA Model for Predicting GC‐CY1. A) Flowchart outlining the inclusion and exclusion criteria for patients in the prospective study. B) ROC curves for different prediction models in the prospective validation set. C) Calibration curves for the RSA model in the prospective validation set. D) Radar charts displaying the performance indicators of various prediction models in the prospective validation set. E) Confusion matrices for different prediction models in the prospective validation set. F) Double‐layer concentric circle charts demonstrating the clinical advantages of different prediction models in the prospective validation set. G–I) Comparison of clinical impact curves (CIC) for different models in the prospective validation set. J) Flowchart of the RSA model validation for clinical application by radiologists of varying experience levels. K) Comparison of the diagnostic performance of the clinical feature model among radiologists with different experience levels. L) Comparison of the diagnostic performance of the RSA model among radiologists with different experience levels. M) Comparison of radiologists' diagnostic performance with and without the assistance of the RSA model.

To further validate the clinical applicability and practical value of the RSA model, we registered a prospective clinical trial (NCT 06759467) for re‐evaluation. This trial enrolled 152 gastric cancer patients who met the inclusion criteria at the FHHMU from January to June 2024 (**Figure** **5**A). Detailed clinical characteristics of these patients are presented in Table S9 (Supporting Information). In this prospective study, the RSA model demonstrated superior performance in predicting GC‐CY1 compared to the clinical feature model (AUC = 0.771, 95% CI: 0.658–0.885) and the radiomics feature model (AUC = 0.781, 95% CI: 0.678–0.884), with an AUC of 0.835 (95% CI: 0.726–0.943) (Figure 5B–F, Table S10, Supporting Information). The clinical impact curve further confirmed the RSA model's superior predictive performance for GC‐CY1 (Figure 5G–I).

Additionally, we conducted a crossover trial based on this prospective study to evaluate the performance and auxiliary capabilities of the RSA model (Figure 5J). When diagnosing based on clinical information and CT images, the RSA model was more sensitive than senior radiologists [0.810 (95% CI: 0.771–0.848) versus 0.619 (95% CI: 0.581–0.657), *p* < 0.001] and more accurate [0.809 (95% CI: 0.796–0.823) versus 0.722 (95% CI: 0.686–0.758), *p* = 0.042]. The RSA model also outperformed novice radiologists in sensitivity [0.810 (95% CI: 0.775–0.843) versus 0.546 (95% CI: 0.468–0.623), *p* < 0.001] and accuracy [0.784 (95% CI: 0.763–0.805) versus 0.696 (95% CI: 0.586–0.806), *p* = 0.011] (Figure 5K–M; Table S11, Supporting Information).

### 3.6 Biological Characteristics and Immune Infiltration

**Figure 6:**
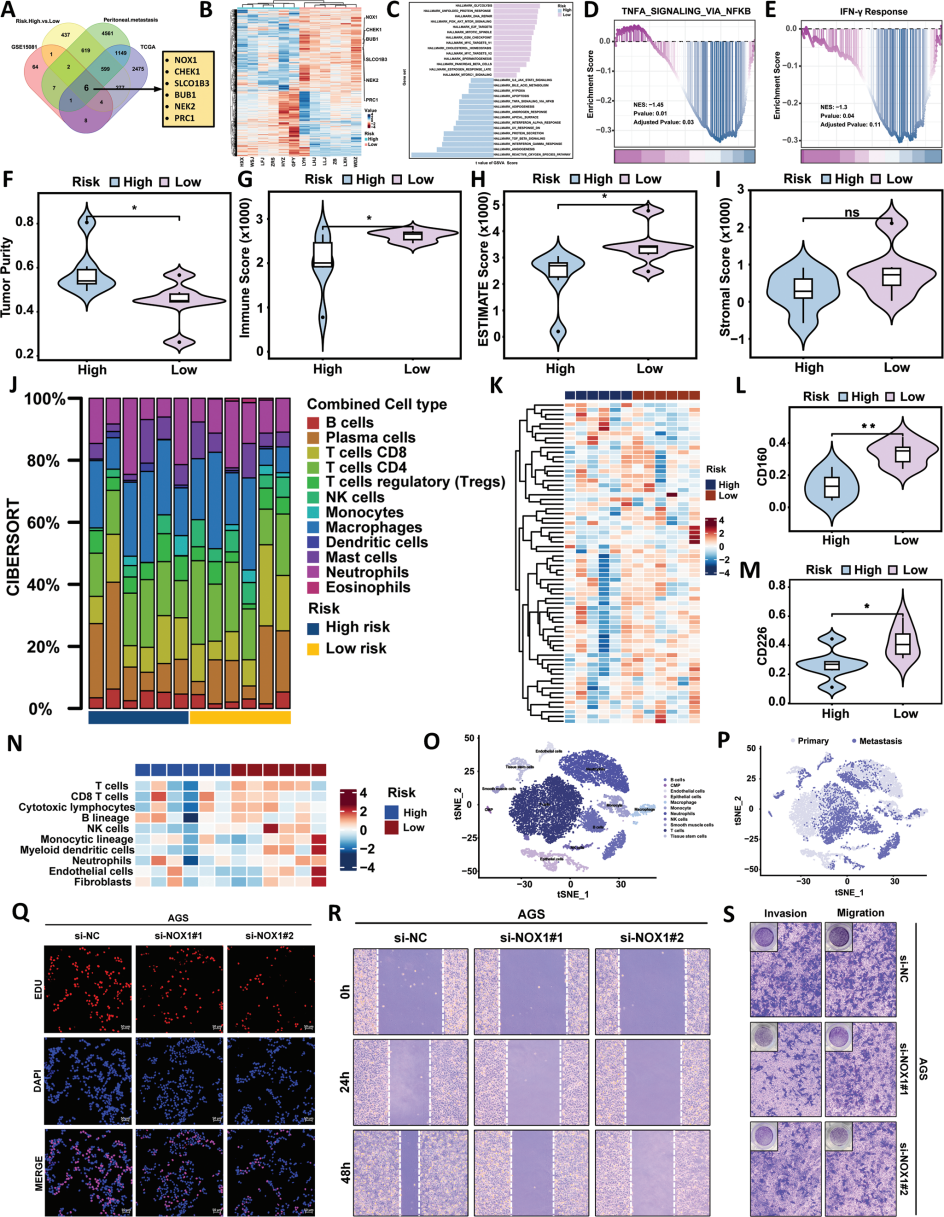
Biological characteristics and immune infiltration in high‐risk and low‐risk groups. A) Using appropriate transcriptome data from the TCGA database, GEO database, and Venn diagrams of mRNA sequencing of 6 high‐risk patients and 6 low‐risk patients, 6 candidate mRNAs were found. B) Heatmap shows the expression of 6 candidate mRNAs in high‐risk and low‐risk patients. C) Gene set variation analysis (GSVA) enrichment analysis shows pathways enriched in high‐risk and low‐risk groups. D,E) Gene set enrichment analysis (GSEA) signature pathways of IFNγ (D) and TNFα (E) were found to be statistically enriched in low‐risk features (*p* < 0.05). F–I) Violin plots show the differences in tumor purity (F), immune score (G), ESTIMATE score (H), and stromal score (I) between high‐risk and low‐risk groups. J) By using the cell‐type Identification by Estimating Relative Subsets of RNA Transcripts (CIBERSORT) algorithm, the scores of combined cell types show the proportional diversity between features. K‐M) Heatmaps (K) and violin plots (L,M) show the expression and differences of immune checkpoint genes in high‐risk and low‐risk patients. N) The relative abundance of each immune cell in tumor tissue was calculated by microenvironment cell populations‐counter (MCPcounter) algorithm and displayed as a heat map. O,P) t‐SNE diagram of cell type (O) and metastasis type (P) from single cell data of gastric cancer patients. Q) EdU experiment to detect DNA replication activity of AGS cells after silencing NOX1. R) Scratch healing assay to detect migration ability of AGS cells after silencing NOX1. S) Transwell assay to detect migration and invasion ability of AGS cells after silencing NOX1.

To explore the immunological characterization of this feature, we performed Gene set variation analysis (GSVA) functional enrichment analysis using RNA sequencing data of gastric cancer samples (6 samples were high‐risk and 6 samples were low‐risk), and found that immune‐related pathways in the low‐risk feature included overall upregulation of interferon (IFN) and upregulation of the TNFα pathway (**Figure** **6**C). In addition, in further Gene set enrichment analysis (GSEA) enrichment analysis, the results also showed that the IFNγ and TNFα pathways were upregulated in the low‐risk feature (Figure 6D,E). Next, we focused on the characterization of immune infiltration in the local immune signaling environment. We calculated the immune infiltration score of each sample through ESTIMATE analysis, and the results showed that the immune and ESTIMATE scores of patients in the low‐risk group were significantly higher than those in the high‐risk group (Figure 6F–I). Since cell types vary with local signaling networks and drive cell activity within tumors, we studied whether cell states and multicellular communities differ between different features. We calculated the relative abundance of each immune cell in tumor tissue using two algorithms, CIRBERSORT and microenvironment cell populations‐counter (MCPcounter). In terms of cell status, both methods showed that the abundance of NK cells in low‐risk features was statistically higher (Figure 6J,N; Figure S6A,D, Supporting Information). In addition, CIRBERSORT showed that the abundance of dendritic cells was increased in high‐risk features (Figure S6B, Supporting Information), while microenvironment cell populations‐counter (MCPcounter) showed that T cells showed that the abundance of dendritic cells was increased in low‐risk features (Figure S6C, Supporting Information). Further immune checkpoint analysis results showed (Figure 6K) that the immune promotion‐related targets CD160 and CD226 were more highly expressed in low‐risk features (Figure 6L,M), while the immune suppression targets CEACAM1 and PVR were significantly increased in high‐risk features (Figure S6E,F, Supporting Information). These results indicate the potential role of immune checkpoint therapy for low‐risk populations.

In order to further explore the biological function that affects this feature, we combined sequencing, The Cancer Genome Atlas (TCGA) and GEO database to screen the genes related to the feature (Figure 6A), and displayed the gene expression by drawing a heat map (Figure 6B). Next, we revealed the potential role of relevant genes in the immune microenvironment through single‐cell transcriptome. We obtained 11 cell subpopulations (Figure 6O,P) through filtering, dimensionality reduction, clustering, and cell grouping of single cell data, and showed the expression of six genes in the immune microenvironment. The results showed that the six genes were mostly expressed in epithelial cells and metastasis groups (Figure S7, Supporting Information). By comparing the degree of differential changes of six genes in high‐ and low‐risk traits, NOX1 had the most significant differential changes, so we selected NOX1 for in vitro experiments. EdU (Figure 6Q; Figure S8A, Supporting Information), scratch healing (Figure 6R; Figure S8B, Supporting Information) and transwell (Figure 6S; Figure S8C,D, Supporting Information) results demonstrated that knockdown of NOX1 functionally promoted AGS cell proliferation, migration and invasion abilities.

## 4 Discussion

This study presents a novel approach for diagnosing peritoneal lavage cytology‐positive gastric cancer (GC‐CY1) using a multimodal artificial intelligence (AI)‐based virtual biopsy model, the Risk Stratification Assessment (RSA) model. The RSA model demonstrated superior performance compared to traditional clinical and radiomics model, achieving high predictive accuracy across multiple cohorts. Specifically, the model achieved an AUC of 0.866 in the training cohort and 0.835 in the prospective validation, with similar results observed in the external validation sets. Additionally, the RSA model showed significant clinical benefits in identifying patients at high risk of recurrence and those who may benefit from specific treatments, such as paclitaxel‐based conversion therapy. These findings suggest that the RSA model can provide a reliable non‐invasive alternative to current diagnostic methods, potentially leading to earlier detection and improved management of GC‐CY1.

Comparatively, previous studies have explored various diagnostic modalities for peritoneal involvement in gastric cancer, including CT imaging, laparoscopic exploration, and the use of radiomic biomarkers.^[^ ^21^, ^22^, ^23^, ^24^ ^]^ Traditional diagnostic tools, such as CT imaging, are commonly used for preoperative staging but have limited sensitivity for detecting early or microscopic peritoneal metastasis. Studies by Wang et al. and others have demonstrated that CT has a high false‐negative rate for small peritoneal nodules or free cancer cells in the peritoneal cavity, often missing early signs of metastasis.^[^ ^25^ ^]^ In contrast, laparoscopic exploration offers higher sensitivity but remains an invasive procedure with inherent risks, including infection and sampling error, as described by Feussner et al.^[^ ^26^ ^]^ Radiomic biomarkers, such as those evaluated by Jiang et al.,^[^ ^27^ ^]^ have shown promise in non‐invasive detection, but their application has been limited by variability in imaging protocols and the lack of standardized methodologies. In this context, the RSA model's ability to integrate multimodal data and provide a comprehensive assessment of cancer status represents a significant advancement over existing diagnostic approaches.

Furthermore, the RSA model compares favorably with other AI‐based diagnostic tools in oncology. For example, Li et al.^[^ ^28^ ^]^ developed an AI model for predicting lymph node metastasis in gastric cancer, achieving an AUC of 0.84, which is lower than the RSA model's performance for GC‐CY1. Similarly, Chen et al.^[^ ^29^ ^]^ reported an AI model for diagnosing peritoneal metastasis in gastric cancer with an AUC of 0.829, again demonstrating that the RSA model achieves higher predictive accuracy. These comparisons underscore the RSA model's robustness in detecting gastric cancer with peritoneal involvement. Additionally, the RSA model's design, which incorporates both radiomic and clinical data, contrasts with previous studies that primarily focused on single‐modality data. This multimodal approach likely contributes to the model's enhanced diagnostic performance by providing a more comprehensive understanding of the tumor's biological and clinical context.

The superior performance of the RSA model may be explained by its integration of radiomic features and clinical variables, capturing the molecular characteristics and microenvironmental factors that underlie GC‐CY1. The molecular analysis in this study revealed that immune‐related pathways, including interferon (IFN) and TNFα signaling, were upregulated in low‐risk patients, suggesting a more robust anti‐tumor immune response. Conversely, high‐risk patients exhibited increased expression of immune suppression targets, such as CEACAM1 and PVR, which may facilitate immune evasion. These findings align with previous reports on the role of immune checkpoint pathways in gastric cancer progression and support the potential of the RSA model to identify patients who may benefit from immunotherapy. Moreover, the presence of higher NK cell abundance and dendritic cell activity in low‐risk patients suggests an active engagement of the innate immune system, which could be leveraged for therapeutic interventions.^[^ ^30^, ^31^, ^32^ ^]^ The identification of NOX1 as a differentially expressed gene further highlights its role in gastric cancer biology, with functional assays indicating that NOX1 promotes cancer cell proliferation, migration, and invasion, making it a potential target for future therapies.^[^ ^33^, ^34^ ^]^

Despite these promising results, the study has several limitations that should be acknowledged. First, the RSA model's performance is partially dependent on the manual selection of tumor regions during image preprocessing. The accuracy and stability of this process can be influenced by the radiologists’ expertise, leading to potential inter‐observer variability and bias. Standardizing tumor region delineation protocols or integrating automated segmentation tools in future work will be essential to minimize this variability and ensure consistent results. Second, while the RSA model was validated across multiple external cohorts, its performance may still vary in different populations or clinical settings. This variation can be attributed to heterogeneity in imaging protocols, treatment strategies, and underlying oncological characteristics of patient populations from different geographic regions. For instance, tumor biology, genetic predispositions, and environmental factors may differ significantly, potentially impacting model predictions. Further validation in larger, more diverse cohorts, including international datasets, is necessary to confirm the generalizability and robustness of the model across different clinical and demographic contexts. Moreover, the reliance on retrospective data for model training and validation may introduce selection and information biases, as data collection is often incomplete or inconsistent. Prospective, multi‐center studies with standardized data acquisition protocols are needed to address these limitations and validate the model in real‐world clinical practice. Lastly, while the RSA model incorporates a broad range of clinical and radiomic features, it currently does not include genetic or epigenetic data, which may provide additional insights into tumor behavior and prognosis. Future research integrating molecular biomarkers with radiomic data could further improve the model's predictive power and clinical utility.

## 5 Conclusion

In conclusion, this study demonstrates that the multimodal AI‐based RSA model is a powerful tool for diagnosing GC‐CY1, offering significant advantages over traditional diagnostic methods. By combining radiomic and clinical data, the model achieves superior predictive performance, providing a non‐invasive, accurate, and rapid alternative for detecting peritoneal involvement in gastric cancer. The findings suggest that the RSA model can enhance clinical decision‐making, allowing for earlier and more targeted interventions, thereby improving patient outcomes. Future studies should focus on validating the model in diverse populations, integrating additional molecular data, and exploring its potential applications in other cancer types.

## Conflict of Interest

The authors declare no conflict of interest.

## Author Contributions

Conception and design was done by Q.Z. and L.M. Administrative support was done by Q.Z. Provision of study materials or patients was done by P.D., J.Y., H.G., H.W., J.W., T.L., J.H., Y.T., P.Y., R.G., L.Z., N.M., X.L., Z.G., L.M., and Q.Z. Collection and assembly of data was done by J.Y., H.G., H.W., and J.W. Data analysis and interpretation was done by P.D., J.Y., H.G., H.W., J.W., and T.L. Manuscript writing was done by P.D., J.Y., H.G., H.W., J.W., and T.L. All author final approval of manuscript.

## Supporting information



Supporting Information

## Data Availability

The data that support the findings of this study are available from the corresponding author upon reasonable request.
