# Supplementary material for: Multimodal Artificial Intelligence‐Based Virtual Biopsy for Diagnosing Abdominal Lavage Cytology‐Positive Gastric Cancer
Source: Adv Sci (Weinh). 2025 Feb 22;12(15):2411490. doi: 10.1002/advs.202411490 (PMC12005817; doi:10.1002/advs.202411490)
Supplement: Supplementary file 1 — Supporting Information [file ADVS-12-2411490-s001.docx]

**SI materials**

**CT scanning protocol**

All CT examinations in medical institutions across Hebei Province were performed using six multidetector CT scanners: three 256-detector CT scanners and three 128-detector CT scanners. The scan parameters were as follows: a tube voltage of 120 kV and tube current controlled by automatic milliampere-second technology. The scanning range extended from the dome of the diaphragm to the pubic symphysis. For enhanced scanning, a nonionic contrast medium with an iodine content of 300 mg/mL was administered at an injection flow rate of 3.0 mL/s, with a total contrast dose of 2 mL/kg body weight. Arterial and venous phase images were acquired at 25 seconds and 70 seconds after injection, respectively. The reconstruction thickness was 1.0 mm.

The CT protocols at Nanjing Jinling Hospital were performed using dual-source spiral CT scanners. The scanning parameters included a tube voltage of 120 kVp, a tube current of 230 mA, a rotation time of 0.5 seconds, and a pitch of 1.2. The contrast infusion volume was 1.5 mL/kg, with a flowa rate of 3–4 mL/s. The venous phase images were acquired at 60 seconds post-injection, and a delayed scan was performed if necessary, with a post-injection delay of 180 seconds. Imaging was conducted with a section thickness of 1.0–1.5 mm, a field of view of 35–50 cm, and a matrix size of 512 × 512.

The CT protocols at Wuhan University Renmin Hospital were performed using a 256-slice CT scanner. The scanning parameters included a tube voltage of 120 kV, a tube current of 200 mA, and a rotation time of 0.5 seconds. For contrast administration, a dosage of 1.5 mL/kg of nonionic contrast medium with an iodine concentration of 300 mg/mL was infused at a rate of 3–4 mL/s. Venous phase images were obtained 70 seconds after contrast injection. Imaging was performed with a slice thickness of 0.625 mm and a matrix size of 512 × 512.

**Radiomic feature engineering**

Features were divided into four parts, namely histogram, texture, filtering, and shape features. We obtained 107 original features initially, namely 18 histogram, 24 Gray Level Co-occurrence Matrix texture, 14 Gray Level Dependence Matrix texture, 16 Gray Level Size Zone Matrix texture (GLSZM), 16 Gray Level Run Length Matrix texture, five Neighborhood Graytone Difference Matrix texture features, and 14 shape features. Subsequently, based on the texture and histogram features, 744 wavelet features were calculated from the configured wavelet filtering and 279 LoG (Laplacian of Gaussian) features were transformed from LoG filtering with 1/2/3 kernel sizes.

**Calculation of peripheral blood inflammatory indexes**

Neutrophil, lymphocyte, platelet, and albumin counts were collected from all patients’ peripheral blood. In this study, SII = neutrophil count × PLT count/lymphocyte count; NLR = neutrophil count/lymphocyte count; PLR = PLT count/lymphocyte count; PNI = Alb concentration + 5 × total lymphocyte count. The cut-off values of the above four variables were calculated and confirmed using X-tile software.

**Assessment of treatment response**

In our study, all initial visits for gastric cancer included abdominal CT scans to assess tumor size[1-2]. Patients were instructed to drink 800-1000 ml of water 30 minutes before the scan, except those with pyloric obstruction. Scopolamine butylbromide was administered intramuscularly to reduce gastrointestinal peristalsis, allowing full gastric lumen dilation and preventing the appearance of gastric wall thickening, thus improving lesion localization and observation. This approach enabled accurate identification of target lesions on CT scans, allowing for precise measurements of tumor diameter and thickness to assess changes in response to neoadjuvant therapy. For each patient, the axial image showing the largest lesion was selected to measure the longest tumor diameter, and perigastric lymph node lesions with a short diameter >1.5 cm were recorded. All measurements were conducted by a senior radiologist. The sum of diameters for all target lesions at baseline (longest diameter for non-lymph node lesions and the shortest axis for lymph node lesions) served as the reference for subsequent evaluations. The final measurement was calculated as the average of three measurements to assess therapeutic efficacy before and after conversion therapy.

Tumor response was evaluated according to the Response Evaluation Criteria in Solid Tumors (RECIST) version 1.1, which classifies responses as complete response (CR), partial response (PR), stable disease (SD), and progressive disease (PD)[3]. CR is defined as the disappearance of all target lesions; PR as a reduction of at least 30% in the sum of the diameters of target lesions from baseline; PD as an increase of at least 20% in the sum of diameters of target lesions from baseline; and SD as a change that does not meet criteria for either PR or PD. The objective response rate (ORR) was the proportion of patients achieving CR and PR, while the disease control rate (DCR) included patients achieving CR, PR, or SD.

Pathological sections from patients who underwent radical surgical resection were reviewed by two pathologists, and tumor regression was graded using the Tumor Regression Grade (TRG) scale according to AJCC/CAP guidelines[1]. TRG 0 is defined as no residual tumor cells in multiple consecutive sections under microscopy; TRG 1 as few discrete tumor cell clusters beneath the plasma membrane; TRG 2 as fibrosis and residual tumor cell debris within the lesion; and TRG 3 as a stable, non-fibrotic cell population within the lesion.

**Diagnostic criteria for tumor cachexia**

The diagnostic criteria for tumor cachexia are as follows [4-5]: (1) Unintentional weight loss of more than 5% within 6 months; (2) Body mass index (BMI) < 20 kg/m² for Europeans and Americans, or BMI < 18.5 kg/m² for Chinese, with any weight loss > 2% within 6 months; (3) Limb skeletal muscle index indicating sarcopenia (male < 7.26 kg/m², female < 5.45 kg/m²) combined with any weight loss > 2% (based on the European Association for the Study of Palliative Care criteria); (4) Presence of reduced food intake and/or systemic inflammation.

**Paclitaxel-based chemotherapy regimen**

ChiCTR1800014817: The first course of hyperthermic intraperitoneal chemotherapy (HIPEC) started on the day after laparoscopic exploration and was performed every 24 h. Three consecutive courses of HIPEC were performed, and the intraperitoneal injection of paclitaxel was chosen (75 mg/m^2^, added to 4,000 mL of 0.9% sodium chloride solution, with a perfusion rate of 500 mL/min, the water temperature was controlled at 43℃, and the perfusion time was 60 min); After 14d of HIPEC treatment, we started to administer abatinib 500 mg/d orally for 21d. At the same time, we administered tiglio 80 mg/(m²·d) orally 30 min after breakfast and dinner for 14d, and then stopped for 7d. Every 3 weeks, we had one course of treatment. The dosage of Tegio was based on body surface area (BSA): 80 mg/ (m²·d) for BSA < 1.25 m^2^, 100 mg/ (m²·d) for BSA 1.25 ~ 1.50 m^2^, and 120 mg/ (m²·d) for BSA > 1.50 m^2^.

NCT03718624: The paclitaxel-based conversion therapy included a combination of intraperitoneal (IP) and intravenous (IV) administration of paclitaxel, alongside oral S-1 and apatinib, as described below: 1) Paclitaxel: IP: 20 mg/m² on days 1 and 8 (dissolved in 1,000 mL saline, infused for >1 hour). IV: 50 mg/m² on days 1 and 8 (dissolved in 500 mL saline, infused for >1 hour). 2) Premedication: Dexamethasone and cimetidine were administered prior to paclitaxel. 3) S-1: Oral administration for 14 consecutive days: BSA <1.25 m²: 80 mg/(m²·d); BSA 1.25–1.50 m²: 100 mg/(m²·d); BSA >1.50 m²: 120 mg/(m²·d). 4) Apatinib: 500 mg/day orally for 21 consecutive days. Each treatment course lasted 3 weeks, and patients underwent CT imaging following two cycles of preoperative chemotherapy to assess efficacy and resectability.

**RNA sequencing**

We isolated and purified RNA from total samples using TRIzol (thermofisher, 15596018) according to the manufacturer's protocol. The quantity and purity of total RNA were then controlled using NanoDrop ND-1000 (NanoDrop, Wilmington, DE, USA), and the integrity of RNA was tested using Bioanalyzer 2100 (Agilent, CA, USA); the concentration was >50 ng/μL, the RIN value was >7.0, and the total RNA was >1 μg, which was sufficient for downstream experiments. Oligo(dT) magnetic beads (Dynabeads Oligo(dT), cat.25-61005, Thermo Fisher, USA) were used for two rounds of purification to specifically capture mRNA with PolyA, and then fragmented using a magnesium ion fragmentation kit (NEBNextR Magnesium RNA Fragmentation Module, cat.E6150S, USA) at 94°C for 5-7 minutes. The fragmented RNA was synthesized into cDNA using reverse transcriptase (Invitrogen SuperScriptTM II Reverse Transcriptase, cat.1896649, CA, USA), and then double-stranded synthesis was performed using E. coli DNA polymerase I (NEB, cat.m0209, USA) and RNase H (NEB, cat.m0297, USA) to convert the DNA and RNA complex double-stranded into DNA double-stranded. At the same time, dUTP Solution (Thermo Fisher, cat. R0133, CA, USA) was added to the second strand to make the double-stranded DNA ends blunt, and then an A base was added to each end to enable it to be connected to the adapter with a T base at the end, and the size of the fragment was screened and purified using magnetic beads. The second strand was digested with UDG enzyme (NEB, cat. m0280, MA, US), and then PCR was performed -95℃ pre-denaturation for 3 min, 98℃ denaturation for 8 cycles of 15 s each, 60℃ annealing for 15 s, 72℃ extension for 30 s, and finally 72℃ extension for 5 min to form a library with a fragment size of 300 bp ± 50 bp (strand-specific library). Finally, we used Illumina NovaseqTM 6000 (Hangzhou LC Biotechnology Co., Ltd.) for double-end sequencing according to standard operations, and the sequencing mode was PE150.

**Cell Culture**

AGS cells (Haixing Biotechnology, Suzhou), verified to be mycoplasma-free, were cultured in F-12 medium containing 10% fetal bovine serum (FBS), 100 U/mL penicillin, and 100 μg/mL streptomycin (GIBCO, USA). The cells were maintained at 37°C in a 5% CO₂ humidified incubator.

**Wound Healing Assay**

The wound-healing assay was performed on confluent AGS cell monolayers following si-NOX1 transfection. A linear scratch was carefully introduced using the tip of a sterile 10 μL pipette, ensuring consistent width and minimal disruption to surrounding cells. The cells were then washed twice with PBS to remove any detached debris and cultured in serum-free medium to inhibit cell proliferation. Images of the scratch area were captured at 0 hours using an inverted microscope to establish a baseline. Subsequent observations were conducted at 24 and 48 hours to monitor cell migration into the scratch area. Image analysis software was used to quantify the rate of cell movement, normalized against the initial 0-hour reference.

**Transwell Migration and Invasion Assays**

The Transwell migration and invasion assays were conducted using 24-well Transwell chambers. For the migration assay, the upper chamber membranes were left uncoated, while for the invasion assay, membranes were pre-coated with a thin layer of Matrigel to simulate the extracellular matrix. Following si-NOX1 transfection, AGS cells (4 × 10⁴ per well) were suspended in 200 μL serum-free medium and seeded into the upper chamber. The lower chamber was filled with 600 μL medium containing 10% FBS as a chemoattractant. The cells were incubated at 37°C with 5% CO₂ for 24 to 48 hours. After incubation, non-migrated or non-invaded cells on the upper side of the membrane were gently removed using a cotton swab. The cells that migrated or invaded to the lower side of the membrane were fixed with 4% paraformaldehyde, stained with crystal violet, and washed with PBS. Images were captured, and the number of cells was quantified by counting five randomly selected fields under a microscope.

**Statistical analysis**

The categorical variables were summarized as frequencies and percentages and were compared with the Chi-square test or Fisher’s exact test. The continuous variables were reported as medians with interquartile ranges and were compared with the Mann-Whitney U test. Missing data were handled using multiple imputation by chained equations (MICE) via the “mice” package in R. LASSO was adopted for feature reduction by the “glmnet” package. Univariate Logistic regression analysis was applied to distinguish candidate factors associated with GC-CY1, and multivariate Logistic regression analysis was performed to select independent prognostic predictors. The Kaplan-Meier curves and the log-rank test were used to estimate the survival rates of different patient groups. The hazard ratio (HR) was calculated by Logistic regression analysis. In this study, three nomograms were developed. The agreement between the actual survival rates and the predicted probability of nomograms was compared by calibration curves. The “rms” package was used for the Logistic regression analyses, nomograms, and calibration plots. To evaluate the predictive performance of nomograms, we calculated the area under the receiver operating characteristic (ROC) curve (AUC) at given time points. The "pROC" package was used to evaluate whether the improvements in AUCs between logistic regression models for prediction were statistically significant. Analyses were conducted in R with two-tailed tests. A P value of < .05 was deemed statistically significant.

**References**

Ding P, Yang J, Wu J, Wu H, Sun C, Chen S, Yang P, Tian Y, Guo H, Liu Y, Meng L, Zhao Q. Combined systemic inflammatory immune index and prognostic nutrition index as chemosensitivity and prognostic markers for locally advanced gastric cancer receiving neoadjuvant chemotherapy: a retrospective study. BMC Cancer. 2024 Aug 15;24(1):1014. doi: 10.1186/s12885-024-12771-z.

Ding P, Yang P, Sun C, Tian Y, Guo H, Liu Y, Li Y, Zhao Q. Predictive Effect of Systemic Immune-Inflammation Index Combined With Prognostic Nutrition Index Score on Efficacy and Prognosis of Neoadjuvant Intraperitoneal and Systemic Paclitaxel Combined With Apatinib Conversion Therapy in Gastric Cancer Patients With Positive Peritoneal Lavage Cytology: A Prospective Study. Front Oncol. 2022 Jan 19;11:791912. doi: 10.3389/fonc.2021.791912.

Jang GS, Kim MJ, Ha HI, Kim JH, Kim HS, Ju SB, Zang DY. Comparison of RECIST version 1.0 and 1.1 in assessment of tumor response by computed tomography in advanced gastric cancer. Chin J Cancer Res. 2013 Dec;25(6):689-94. doi: 10.3978/j.issn.1000-9604.2013.11.09.

1. Fearon K, Strasser F, Anker SD, Bosaeus I, Bruera E, Fainsinger RL, Jatoi A, Loprinzi C, MacDonald N, Mantovani G, Davis M, Muscaritoli M, Ottery F, Radbruch L, Ravasco P, Walsh D, Wilcock A, Kaasa S, Baracos VE. Definition and classification of cancer cachexia: an international consensus. Lancet Oncol. 2011 May;12(5):489-95. doi: 10.1016/S1470-2045(10)70218-7.
2. Baracos VE, Mazurak VC, Bhullar AS. Cancer cachexia is defined by an ongoing loss of skeletal muscle mass. Ann Palliat Med. 2019 Jan;8(1):3-12. doi: 10.21037/apm.2018.12.01.

**SI Figures**

**
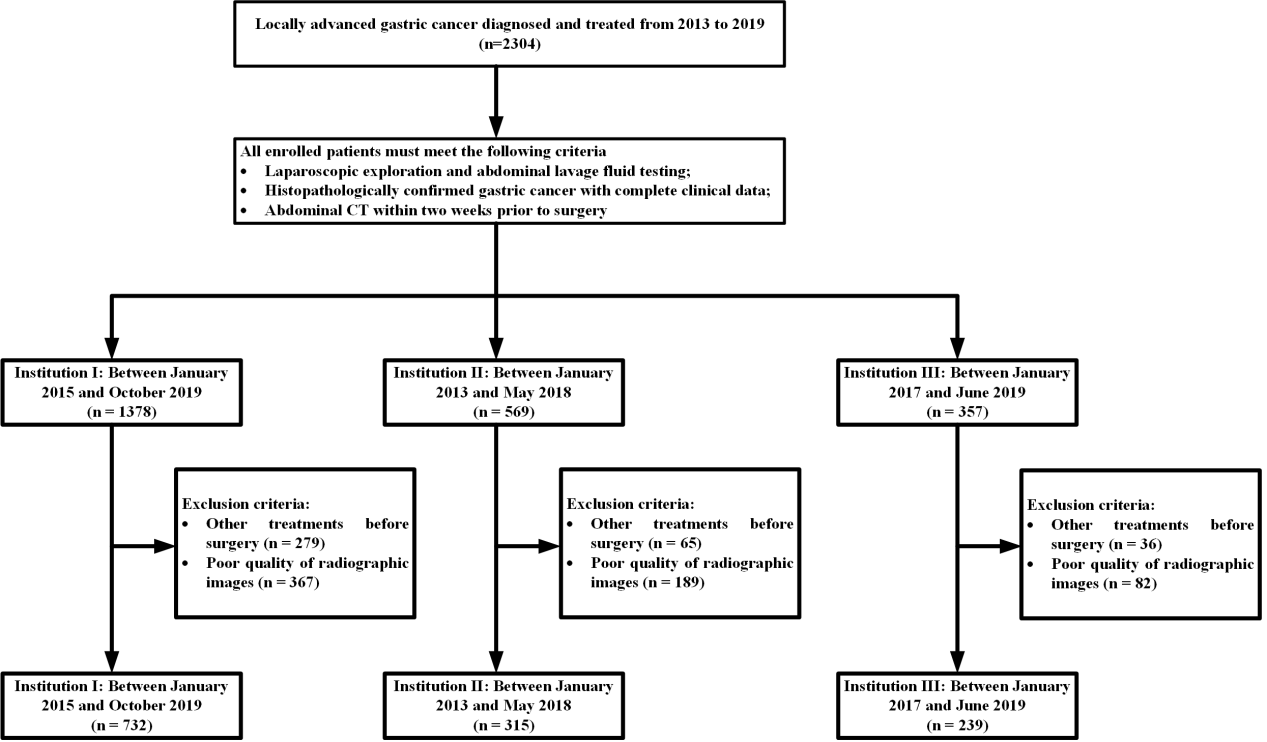
**

**Figure S1. Flowchart of inclusion and exclusion of patients in the training set and external validation set.**

**
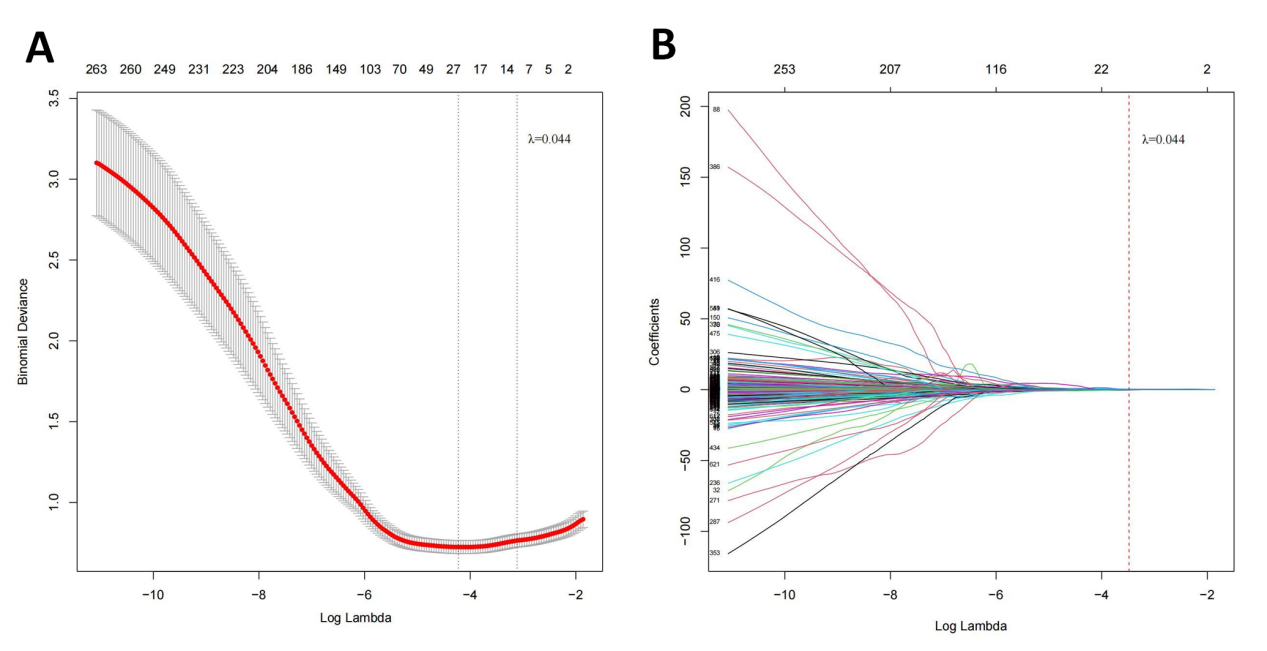
**

**Figure S2. LASSO for feature reduction in the training cohort.** We used LASSO for feature reduction and to select the candidate features. (A) The optimal penalization coefficient lambda (λ) in the LASSO model using ten-fold cross-validation was identified. To avoid overfitting, we used the 1-standard error criterion (λ=0.044) with log (λ)= -1.353 to determine the number of selected features. (B) LASSO coefficient profiles of 732 features and the optimal λ resulted in 10 non-zero coefficients, including log-sigma-1-0-mm-3D firstorder Skewness, log-sigma-3-0-mm-3D glszm GrayLevelNonUniformity, wavelet-LLH firstorder Skewness, wavelet-LLH gldm GrayLevelNonUniformity, wavelet-LLH gldm LargeDependenceHighGrayLevelEmphasis, wavelet-LHL firstorder TotalEnergy, wavelet-LHH gldm GrayLevelNonUniformity, wavelet-HLL gldm DependenceNonUniformity, wavelet-HLL glrlm GrayLevelNonUniformity, wavelet-HLH firstorder Kurtosis.


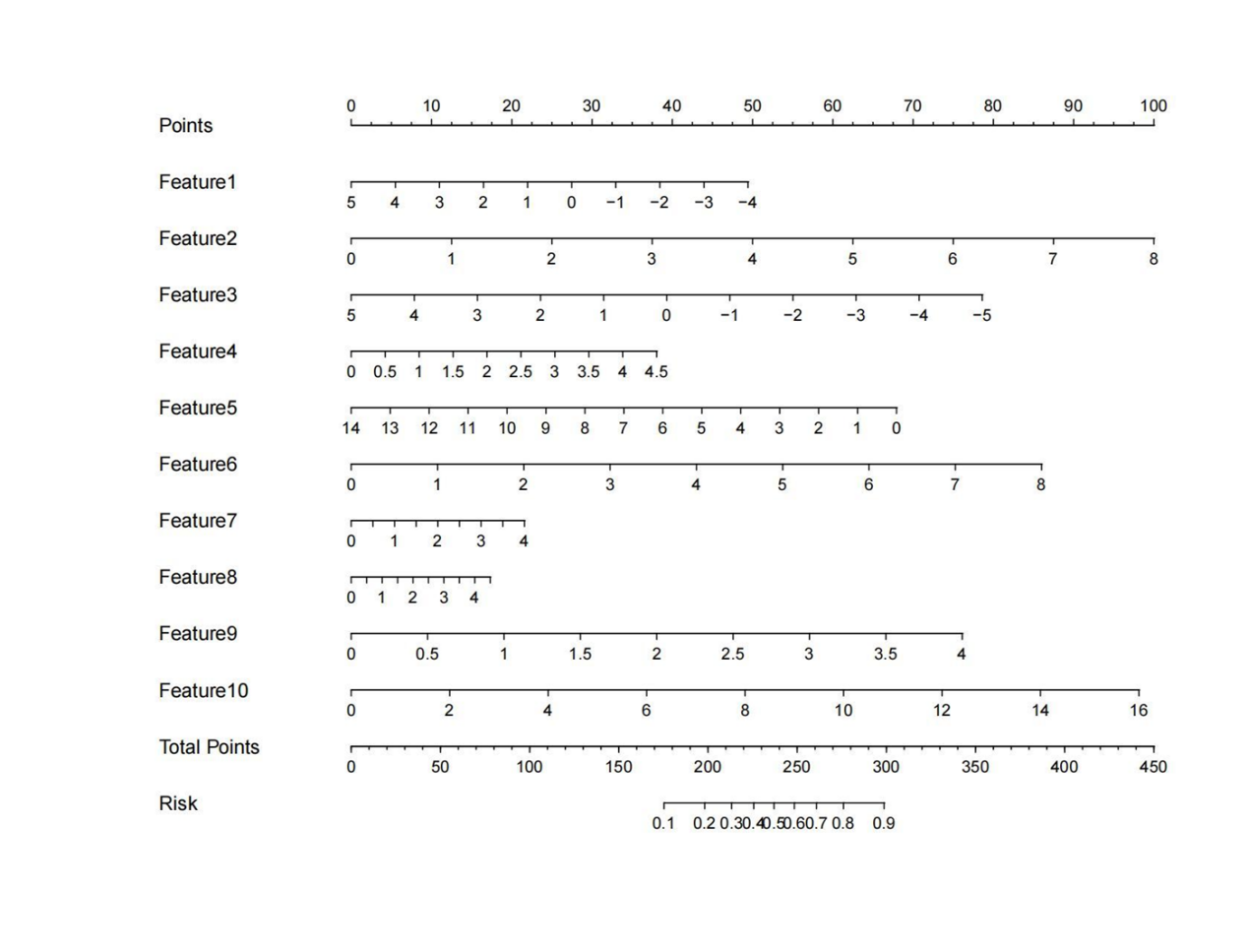


**Figure S3. Visual nomogram for predicting CY1-positivity using radiomics features.**

**
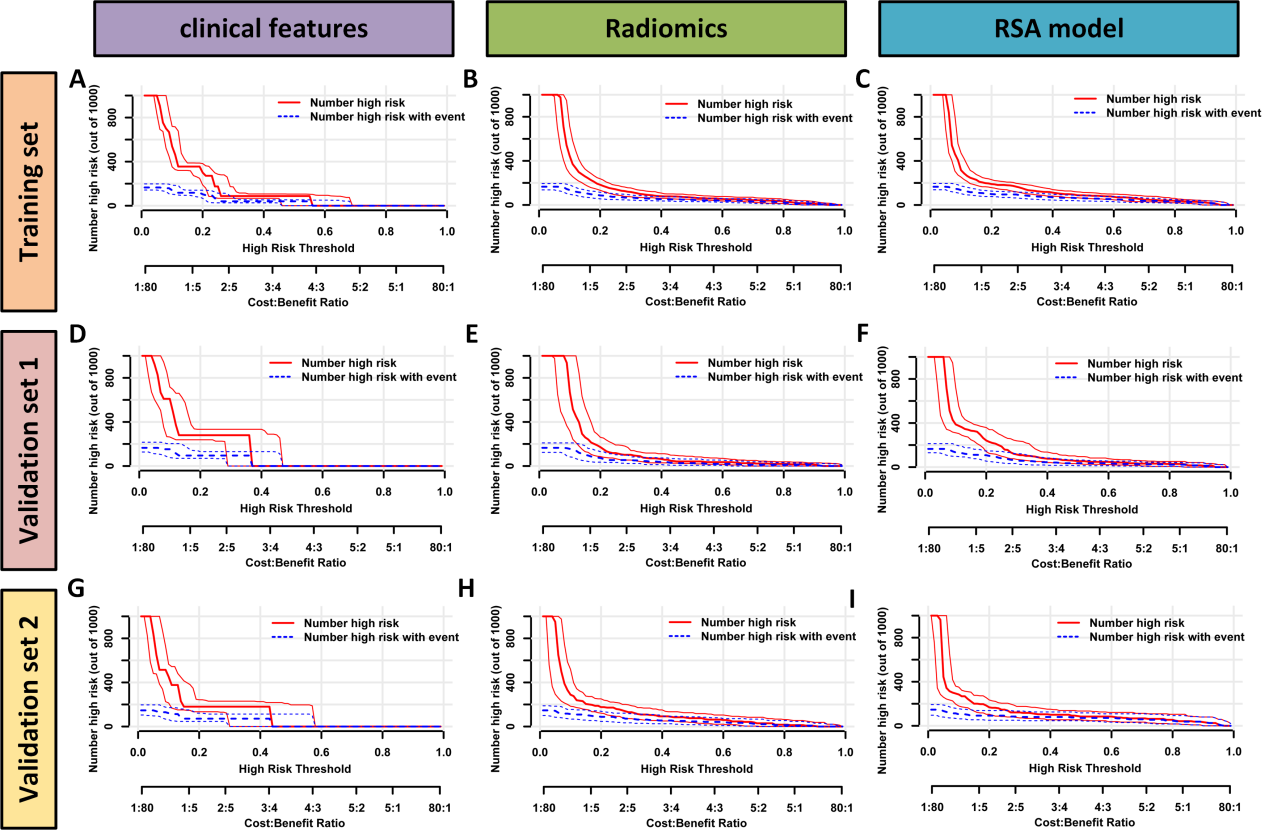
**

**Figure S4. Comparison of clinical impact curves between different models in the training set and external validation set.** (A-C) Clinical impact curves of the clinical feature model (A), radiomics feature model (B), and RSA model (C) in the training set. (D-F) Clinical impact curves of the clinical feature model (D), radiomics feature model (E), and RSA model (F) in the external validation set I. (G-I) Clinical impact curves of the clinical feature model (G), radiomics feature model (H), and RSA model (I) in the external validation set I.


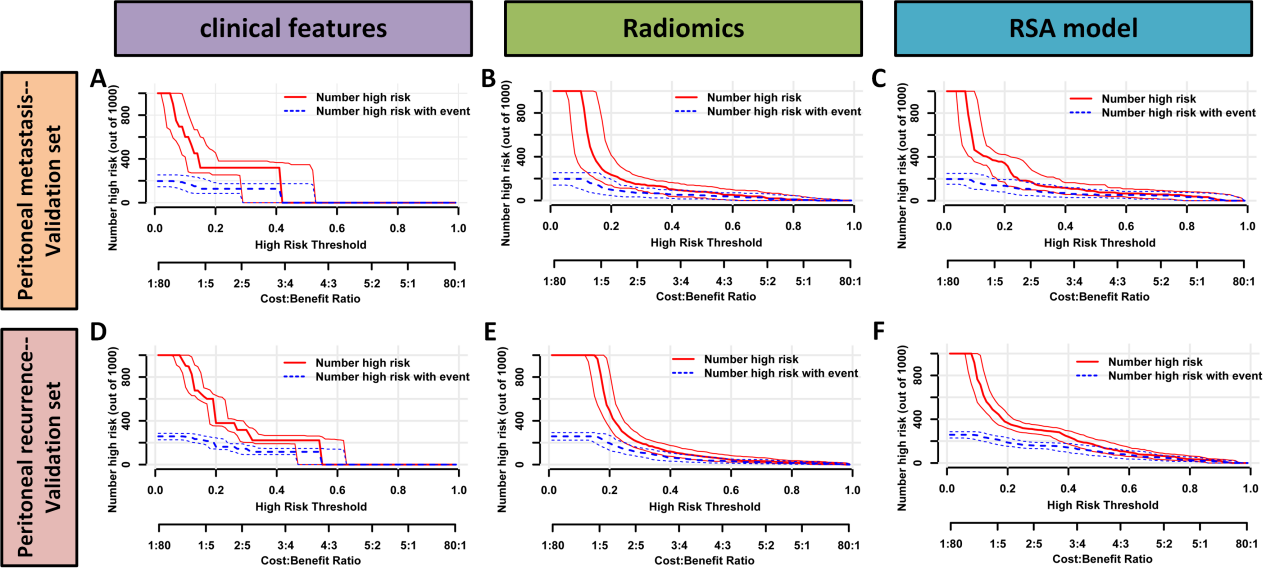


**Figure S5. Comparison of clinical impact curves between different models in the peritoneal metastasis validation set and peritoneal recurrence validation set.** (A-C) Clinical impact curves of the clinical feature model (A), radiomics feature model (B), and RSA model (C) in the peritoneal metastasis validation set. (D-F) Clinical impact curves of the clinical feature model (D), radiomics feature model (E), and RSA model (F) in the peritoneal recurrence validation set.


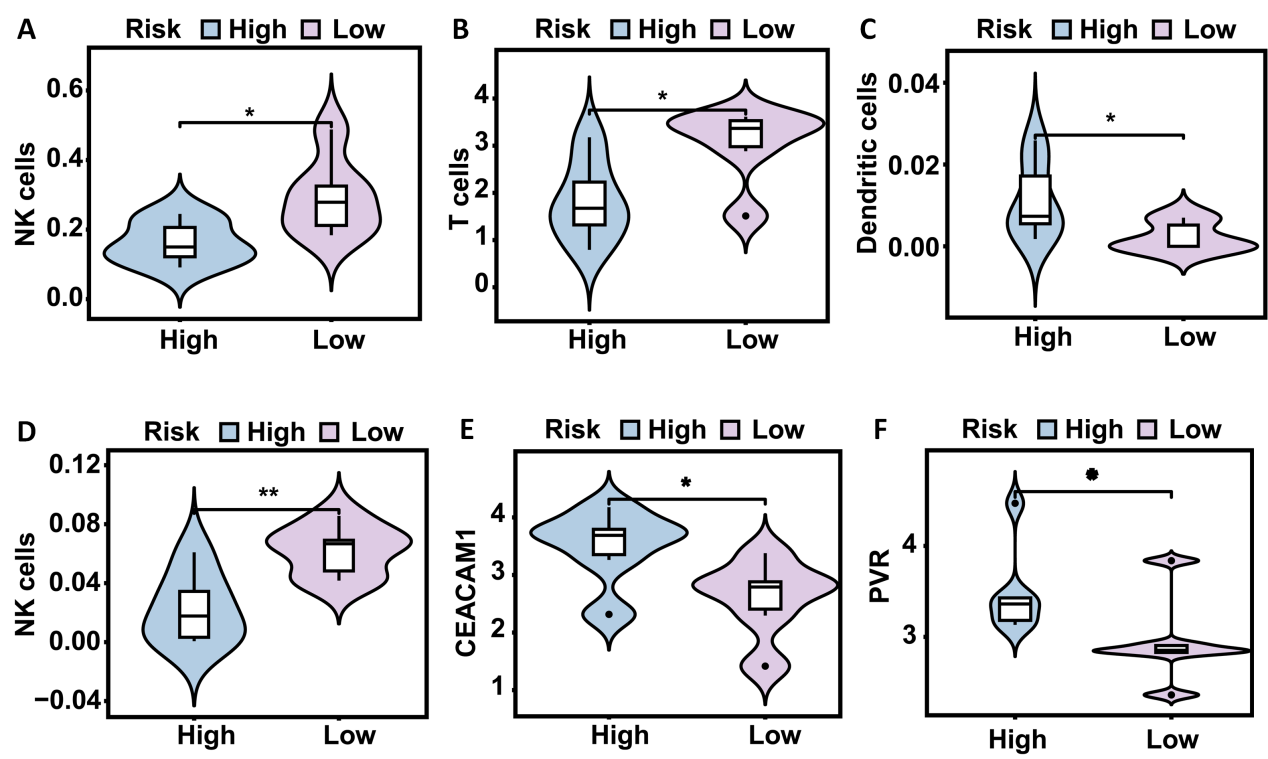


**Figure S6. Immunological differences between high-risk and low-risk groups.** (A-B). After calculating the relative abundance of each immune cell in tumor tissue using the MCPcounter algorithm, the violin plots show the differences in NK cells (A) and T cells (B) between high-risk and low-risk patients. (C-D). After calculating the relative abundance of each immune cell in tumor tissue using the CIBERSORT algorithm, the violin plots show the differences in Dendritic cells (C) and NK cells (D) between high-risk and low-risk patients. (E-F). Differences in the expression of immune checkpoints CEACAM1 (E) and PVR (F) between high-risk and low-risk patients.


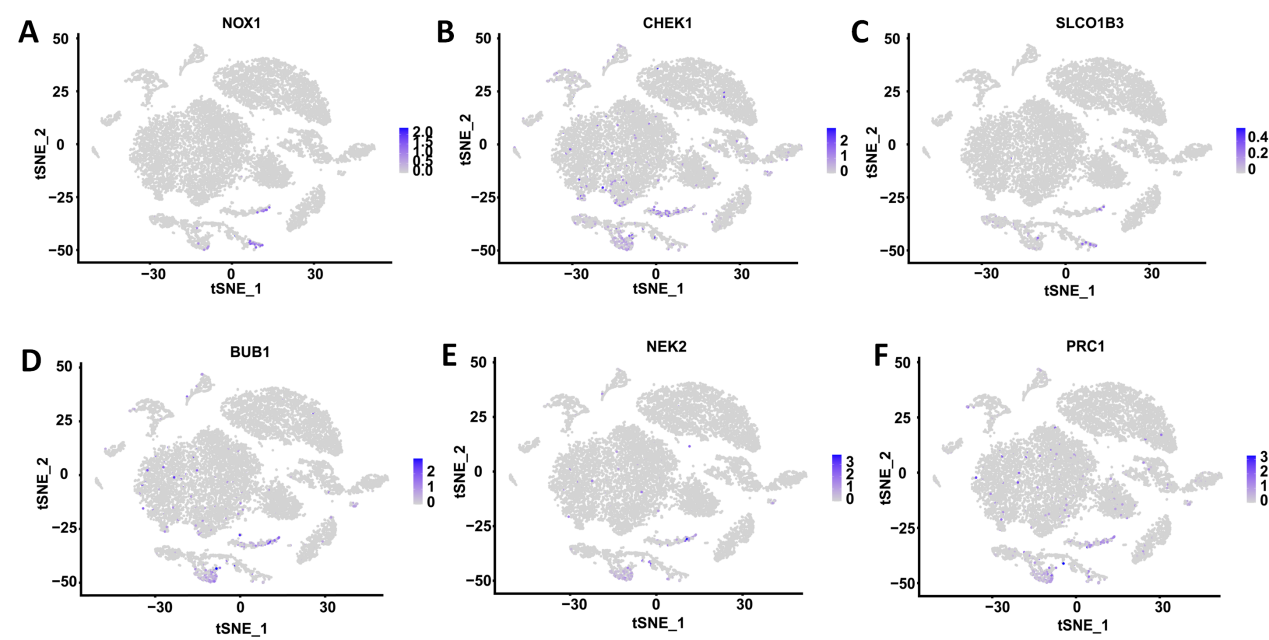


**Figure S7. Expression distribution of six candidate genes (NOX1, CHEK1, SLCO1B3, BUB1, NEK2, and PRC1) in the tumor microenvironment.**


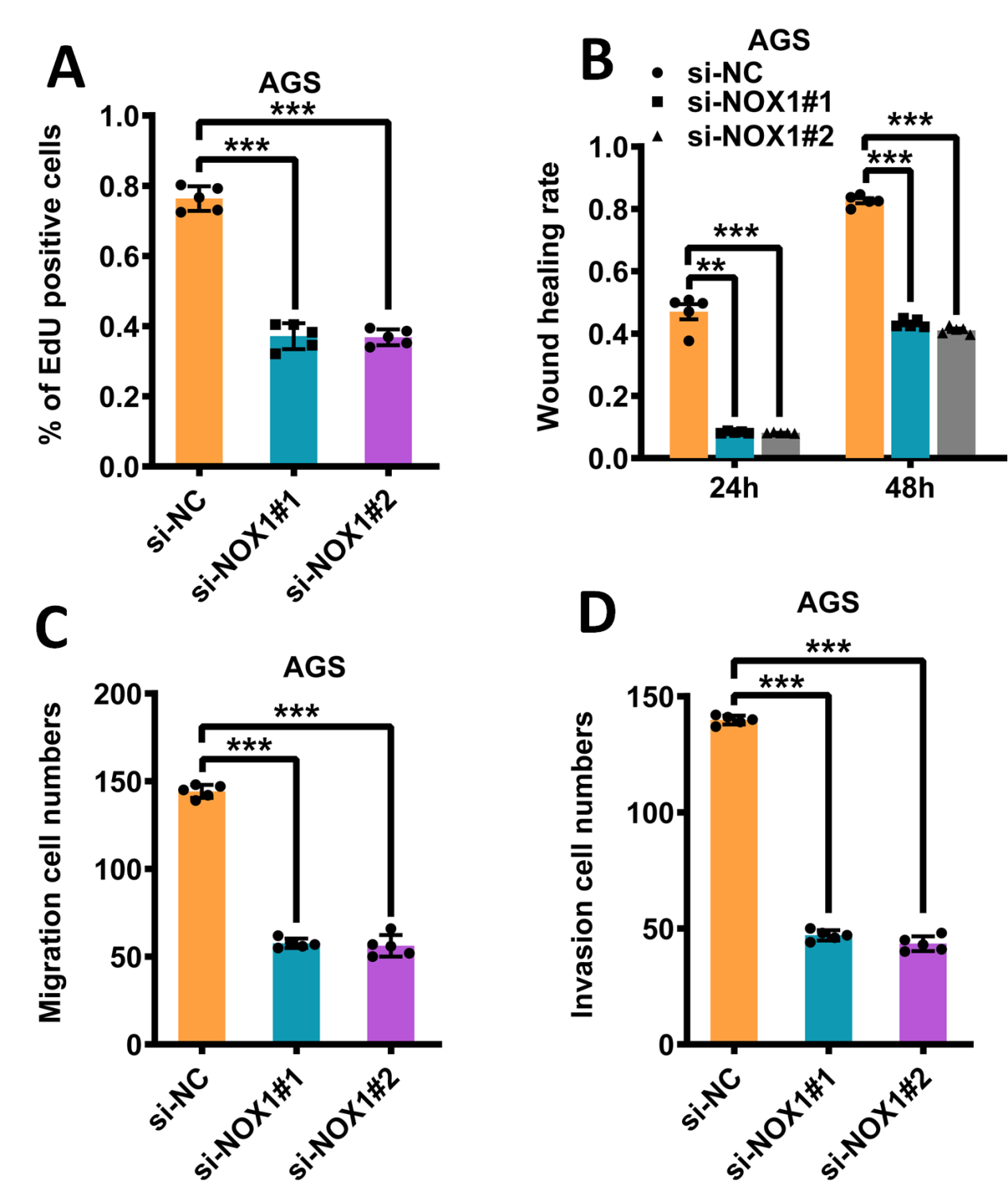


**Figure S8. Statistical quantification of the effects of NOX1 on the proliferation, invasion and migration of AGS cells.** A: EdU assay in AGS cells verified the statistical graph of NOX1 proliferative ability. B: Statistical graph of NOX1 migration ability verified by wound healing assay in AGS cells. C-D: Statistical graph of NOX1 migration (C) and invasion (D) ability verified by Transwell assay in AGS cells.

**SI Tables**

**Table S1. Medical center information included in the study**

| **Center Number** | **Medical Center Name** | **City** | **Province** | **Patient Cohort** | **Number of Patients** |
| --- | --- | --- | --- | --- | --- |
| 1 | The Fourth Hospital of Hebei Medical University | Shijiazhuang | Hebei | training set | 546 |
| 2 | Shijiazhuang People's Hospital | Shijiazhuang | Hebei | training set | 186 |
| 3 | Baoding Central Hospital | Baoding | Hebei | validation set I | 177 |
| 4 | Hengshui People's Hospital | Hengshui | Hebei | validation set I | 138 |
| 5 | Jinling Hospital, Medical School of Nanjing University | Nanjing | Jiangsu | validation set Ⅱ | 136 |
| 6 | Wuhan University People's Hospital | Wuhan | Hubei | validation set Ⅱ | 103 |

**Table S2. Multivariate analysis of radiomics characteristics affecting CY1-positivity in LAGC patients**

| **Radiomics features** | **coefficient estimates** | **95%CI** | **P value** |
| --- | --- | --- | --- |
| **log-sigma-1-0-mm-3D firstorder Skewness** | -0.196 | -0.461 to -0.070 | 0.019 |
| **log-sigma-3-0-mm-3D glszm GrayLevelNonUniformity** | 1.445 | 1.124 to 2.014 | 0.026 |
| **wavelet-LLH firstorder Skewness** | -0.280 | -0.565 to -0.004 | 0.004 |
| **wavelet-LLH gldm GrayLevelNonUniformity** | 1.301 | 1.073 to 2.076 | 0.033 |
| **wavelet-LLH gldm LargeDependenceHighGrayLevelEmphasis** | -0.173 | -0.358 to -0.013 | 0.029 |
| **wavelet-LHL firstorder TotalEnergy** | 1.383 | 1.068 to 2.834 | 0.016 |
| **wavelet-LHH gldm GrayLevelNonUniformity** | 1.692 | 1.216 to 2.700 | 0.011 |
| **wavelet-HLL gldm DependenceNonUniformity** | 1.137 | 1.052 to 2.326 | 0.016 |
| **wavelet-HLL glrlm GrayLevelNonUniformity** | 1.678 | 1.117 to 2.773 | 0.026 |
| **wavelet-HLH firstorder Kurtosis** | 1.219 | 1.055 to 2.492 | 0.006 |

**Table S3. Multivariate logistic regression analysis of peritoneal free cancer cells (GC-CY1) in patients with gastric cancer**

| **Training cohort** | | | |
| --- | --- | --- | --- |
| **Variables** | **OR** | **95%CI** | **P value** |
| T stage (T4 vs. T2/T3) | 2.993 | 1.542-5.809 | 0.001 |
| N stage (N+ vs. N0) | 2.197 | 1.194-4.043 | 0.011 |
| Pathological type (Low/None vs. High/Median) | 2.760 | 1.433-5.314 | 0.002 |
| SII (High vs. Low) | 5.108 | 3.109-8.644 | ＜0.001 |
| Radiomics (High vs. Low) | 9.206 | 5.620-15.080 | ＜0.001 |
| **Validation cohort I** | | | |
| **Variables** | **OR** | **95%CI** | **P value** |
| T stage (T4 vs. T2/T3) | 3.357 | 1.358-8.295 | 0.009 |
| N stage (N+ vs. N0) | 2.228 | 1.881-5.663 | 0.019 |
| Pathological type (Low/None vs. High/Median) | 5.347 | 1.174-24.342 | 0.030 |
| SII (High vs. Low) | 2.811 | 1.179-6.698 | 0.020 |
| Radiomics (High vs. Low) | 17.830 | 5.321-59.745 | ＜0.001 |
| **Validation cohort II** | | | |
| **Variables** | **OR** | **95%CI** | **P value** |
| T stage (T4 vs. T2/T3) | 5.060 | 1.342-19.080 | 0.017 |
| N stage (N+ vs. N0) | 3.668 | 1.051-12.798 | 0.042 |
| Pathological type (Low/None vs. High/Median) | 6.851 | 1.569-29.919 | 0.011 |
| SII (High vs. Low) | 4.688 | 1.641-13.389 | 0.004 |
| Radiomics (High vs. Low) | 28.165 | 9.798-80.962 | ＜0.001 |
| **Predicting peritoneal metastasis--validation cohort** | | | |
| **Variables** | **OR** | **95%CI** | **P value** |
| T stage (T4 vs. T2/T3) | 3.347 | 1.880-12.734 | 0.036 |
| N stage (N+ vs. N0) | 6.637 | 1.804-24.413 | 0.004 |
| Pathological type (Low/None vs. High/Median) | 3.374 | 1.027-11.081 | 0.045 |
| SII (High vs. Low) | 2.757 | 1.044-7.278 | 0.041 |
| Radiomics (High vs. Low) | 8.500 | 3.593-20.109 | ＜0.001 |
| **Predicting peritoneal recurrence--validation cohort** | | | |
| **Variables** | **OR** | **95%CI** | **P value** |
| T stage (T4 vs. T2/T3) | 3.018 | 1.743-5.223 | ＜0.001 |
| N stage (N+ vs. N0) | 2.561 | 1.480-4.432 | 0.001 |
| Pathological type (Low/None vs. High/Median) | 2.135 | 1.220-3.736 | 0.008 |
| SII (High vs. Low) | 4.209 | 2.712-6.532 | ＜0.001 |
| Radiomics (High vs. Low) | 6.142 | 3.963--9.520 | ＜0.001 |
| **Prospective trial (NCT 06759467)--validation cohort** | | | |
| **mRNA** | **OR** | **95%CI** | **P value** |
| T stage (T4 vs. T2/T3) | 1.620 | 1.078-6.944 | 0.036 |
| N stage (N+ vs. N0) | 2.671 | 1.679-10.501 | 0.014 |
| Pathological type (Low/None vs. High/Median) | 2.877 | 1.375-11.253 | 0.029 |
| SII (High vs. Low) | 4.377 | 1.362-14.065 | 0.013 |
| Radiomics (High vs. Low) | 7.399 | 2.342-23.379 | 0.001 |

**Note:** SII = Systemic Immune-inflammation Index; OR = odds ratio.

**Table S4. Comparison of performance indicators of different models for predicting the GC-CY1**

| **Variable** | **AUC** | **Accuracy** | **Sensitivity** | **Specificity** | **PPV** | **NPV** | **PLR** | **NLR** | **F1 score** |
| --- | --- | --- | --- | --- | --- | --- | --- | --- | --- |
| **Training set** |  |  |  |  |  |  |  |  |  |
| Clinical features | 0.751 | 0.758 | 0.537 | 0.802 | 0.349 | 0.897 | 2.713 | 0.577 | 0.423 |
| Radiomics | 0.820 | 0.724 | 0.777 | 0.713 | 0.349 | 0.941 | 2.712 | 0.313 | 0.482 |
| RSA model | 0.866 | 0.848 | 0.603 | 0.897 | 0.537 | 0.919 | 5.851 | 0.442 | 0.568 |
| **Validation set I** |  |  |  |  |  |  |  |  |  |
| Clinical features | 0.791 | 0.746 | 0.577 | 0.779 | 0.341 | 0.903 | 2.616 | 0.543 | 0.429 |
| Radiomics | 0.799 | 0.629 | 0.942 | 0.567 | 0.301 | 0.980 | 2.173 | 0.102 | 0.456 |
| RSA model | 0.883 | 0.784 | 0.750 | 0.791 | 0.415 | 0.941 | 3.587 | 0.316 | 0.534 |
| **Validation set II** |  |  |  |  |  |  |  |  |  |
| Clinical features | 0.757 | 0.816 | 0.486 | 0.873 | 0.395 | 0.908 | 3.811 | 0.589 | 0.436 |
| Radiomics | 0.799 | 0.854 | 0.743 | 0.873 | 0.500 | 0.952 | 5.829 | 0.295 | 0.598 |
| RSA model | 0.832 | 0.891 | 0.743 | 0.917 | 0.605 | 0.954 | 8.914 | 0.281 | 0.667 |

Note: AUC, area under the curve; PPV, Positive Predictive Value; NVP, Negative Predictive Value; PLR, Positive Likelihood Ratio; NLR, Negative Likelihood Ratio.

**Table S5. Clinical characteristics of the conversion therapy patient cohort with GC-CY1 [n(%)]**

| **Clinical characteristic** | **Validation cohort I (NCT03718624)**  **(N=36)** | **Validation cohort II (ChiCTR1800014817)**  **(N=38)** | **P value** |
| --- | --- | --- | --- |
| **Gender** |  |  | 0.778 |
| Male | 22 (61.1%) | 22 (57.9%) |  |
| Female | 14 (38.9%) | 16 (42.1%) |  |
| **Age (years)** |  |  | 0.665 |
| ≤65 | 20 (55.6%) | 23 (60.5% ) |  |
| ＞65 | 16 (44.4%) | 15 (39.5%) |  |
| **T stage** |  |  | 0.197 |
| T2/T3 | 10 (27.8%) | 16 (42.1%) |  |
| T4 | 26 (72.2%) | 22 (57.9%) |  |
| **N stage** |  |  | 0.911 |
| N0 | 7 (19.4%) | 7 (18.4%) |  |
| N+ | 29 (80.6%) | 31 (81.6%) |  |
| **Primary site** |  |  | 0.859 |
| Up 1/3 | 12 (33.3%) | 12 (31.6%) |  |
| Middle 1/3 | 11 (30.6%) | 10 (26.3%) |  |
| Lower 1/3 | 13 (36.1%) | 16 (42.1%) |  |
| **Tumor size (cm)** |  |  | 0.386 |
| ≤5 | 9 (25.0%) | 13 (34.2%) |  |
| ＞5 | 27 (75.0%) | 25 (65.8%) |  |
| **Histology** |  |  | 0.919 |
| None/Low | 30 (83.3%) | 32 (84.2%) |  |
| High/Median | 6 (16.7%) | 6 (15.8%) |  |
| **SII** |  |  | 0.880 |
| Low | 25 (69.4%) | 27 (71.1%) |  |
| High | 11 (30.6%) | 11 (28.9%) |  |
| **PNI** |  |  | 0.970 |
| Low | 21 (58.3%) | 22 (57.9%) |  |
| High | 15 (41.7%) | 16 (42.1%) |  |
| **NLR** |  |  | 0.472 |
| Low | 15 (41.7%) | 19 (50.0%) |  |
| High | 21 (58.3%) | 19 (50.0%) |  |
| **PLR** |  |  | 0.970 |
| Low | 21 (58.3%) | 22 (57.9%) |  |
| High | 15 (41.7%) | 16 (42.1%) |  |

**Note:** SII = Systemic Immune-inflammation Index; PNI = Prognostic Nutritional Index; NLR = Neutrophil to Lymphocyte Ratio; PLR = Platelet to Lymphpcyte Ratio.

**Table S6. Clinical characteristics of the validation cohort of patients with gastric cancer peritoneal metastasis [n(%)]**

| **Clinical characteristic** | **Validation cohort (N=213)** |
| --- | --- |
| **Gender** |  |
| Male | 124 (58.2%) |
| Female | 89 (41.8%) |
| **Age (years)** |  |
| ≤65 | 141 (66.2%) |
| ＞65 | 72 (33.8%) |
| **T stage** |  |
| T2/T3 | 46 (21.6%) |
| T4 | 167 (78.4%) |
| **N stage** |  |
| N0 | 54 (25.4%) |
| N+ | 159 (74.6%) |
| **Primary site** |  |
| Up 1/3 | 80 (37.6%) |
| Middle 1/3 | 32 (15.0%) |
| Lower 1/3 | 101 (47.4%) |
| **Tumor size (cm)** |  |
| ≤5 | 113 (53.1%) |
| ＞5 | 100 (46.9%) |
| **Histology** |  |
| None/Low | 162 (76.1%) |
| High/Median | 51 (23.9%) |
| **SII** |  |
| Low | 77 (36.2%) |
| High | 136 (63.8%) |
| **PNI** |  |
| Low | 31 (14.6%) |
| High | 182 (85.4%) |
| **NLR** |  |
| Low | 130 (61.0%) |
| High | 83 (39.0%) |
| **PLR** |  |
| Low | 116 (54.5%) |
| High | 97 (45.5%) |

**Note:** SII = Systemic Immune-inflammation Index; PNI = Prognostic Nutritional Index; NLR = Neutrophil to Lymphocyte Ratio; PLR = Platelet to Lymphocyte Ratio.

**Table S7. Clinical characteristics of the validation cohort of patients with peritoneal recurrence after radical gastrectomy [n(%)]**

| **Clinical characteristic** | **Validation cohort (N=609)** |
| --- | --- |
| **Gender** |  |
| Male | 464 (76.2%) |
| Female | 145 (23.8%) |
| **Age (years)** |  |
| ≤65 | 392 (64.4%) |
| ＞65 | 217 (35.6%) |
| **T stage** |  |
| T2/T3 | 165 (27.1%) |
| T4 | 444 (72.9%) |
| **N stage** |  |
| N0 | 154 (25.3%) |
| N+ | 455 (74.7%) |
| **Primary site** |  |
| Up 1/3 | 226 (37.1%) |
| Middle 1/3 | 92 (15.1%) |
| Lower 1/3 | 291 (47.8%) |
| **Tumor size (cm)** |  |
| ≤5 | 301 (49.4%) |
| ＞5 | 308 (50.6%) |
| **Histology** |  |
| None/Low | 467 (76.7%) |
| High/Median | 142 (23.3%) |
| **SII** |  |
| Low | 328 (53.9%) |
| High | 281 (46.1%) |
| **PNI** |  |
| Low | 85 (14.0%) |
| High | 524 (86.0%) |
| **NLR** |  |
| Low | 383 (62.9%) |
| High | 226 (37.1%) |
| **PLR** |  |
| Low | 308 (50.6%) |
| High | 301 (49.4%) |

**Note:** SII = Systemic Immune-inflammation Index; PNI = Prognostic Nutritional Index; NLR = Neutrophil to Lymphocyte Ratio; PLR = Platelet to Lymphocyte Ratio.

**Table S8. Comparison of performance indicators of different models for predicting peritoneal metastasis and recurrence in patients with gastric cancer**

| **Variable** | **AUC** | **Accuracy** | **Sensitivity** | **Specificity** | **PPV** | **NPV** | **PLR** | **NLR** | **F1 score** |
| --- | --- | --- | --- | --- | --- | --- | --- | --- | --- |
| **Peritoneal metastasis** |  |  |  |  |  |  |  |  |  |
| Clinical features | 0.759 | 0.737 | 0.643 | 0.760 | 0.397 | 0.897 | 2.681 | 0.470 | 0.491 |
| Radiomics | 0.775 | 0.718 | 0.786 | 0.702 | 0.393 | 0.930 | 2.634 | 0.305 | 0.524 |
| RSA model | 0.831 | 0.808 | 0.762 | 0.819 | 0.508 | 0.933 | 4.203 | 0.291 | 0.610 |
| **Peritoneal recurrence** |  |  |  |  |  |  |  |  |  |
| Clinical features | 0.736 | 0.571 | 0.834 | 0.480 | 0.358 | 0.893 | 1.605 | 0.345 | 0.501 |
| Radiomics | 0.737 | 0.694 | 0.701 | 0.692 | 0.442 | 0.869 | 2.278 | 0.432 | 0.542 |
| RSA model | 0.823 | 0.708 | 0.834 | 0.664 | 0.463 | 0.920 | 2.481 | 0.250 | 0.595 |

Note: AUC, area under the curve; PPV, Positive Predictive Value; NVP, Negative Predictive Value; PLR, Positive Likelihood Ratio; NLR, Negative Likelihood Ratio.

**Table S9. Clinical characteristics of the validation cohort of prospective trial (NCT 06759467) [n(%)]**

| **Clinical characteristic** | **Validation cohort (N=152)** |
| --- | --- |
| **Gender** |  |
| Male | 83 (54.6%) |
| Female | 69 (45.4%) |
| **Age (years)** |  |
| ≤65 | 97 (63.8%) |
| ＞65 | 55 (36.2%) |
| **T stage** |  |
| T2/T3 | 35 (23.0%) |
| T4 | 117 (77.0%) |
| **N stage** |  |
| N0 | 52 (34.2%) |
| N+ | 100 (65.8%) |
| **Primary site** |  |
| Up 1/3 | 47 (30.9%) |
| Middle 1/3 | 31 (20.4%) |
| Lower 1/3 | 74 (48.7%) |
| **Tumor size (cm)** |  |
| ≤5 | 80 (52.6%) |
| ＞5 | 72 (47.4%) |
| **Histology** |  |
| None/Low | 127 (83.6%) |
| High/Median | 25 (16.4%) |
| **SII** |  |
| Low | 74 (48.7%) |
| High | 78 (51.3%) |
| **PNI** |  |
| Low | 36 (23.7%) |
| High | 116 (76.3%) |
| **NLR** |  |
| Low | 99 (65.1%) |
| High | 53 (34.9%) |
| **PLR** |  |
| Low | 81 (53.3%) |
| High | 71 (46.7%) |

**Note:** SII = Systemic Immune-inflammation Index; PNI = Prognostic Nutritional Index; NLR = Neutrophil to Lymphocyte Ratio; PLR = Platelet to Lymphocyte Ratio.

**Table S10. Comparison of performance indicators of different models for predicting GC-CY1 in the prospective cohort**

| **Variable** | **AUC** | **Accuracy** | **Sensitivity** | **Specificity** | **PPV** | **NPV** | **PLR** | **NLR** | **F1 score** |
| --- | --- | --- | --- | --- | --- | --- | --- | --- | --- |
| Clinical features | 0.771 | 0.809 | 0.619 | 0.840 | 0.382 | 0.932 | 3.862 | 0.454 | 0.473 |
| Radiomics | 0.781 | 0.717 | 0.762 | 0.710 | 0.296 | 0.949 | 2.627 | 0.335 | 0.427 |
| RSA model | 0.835 | 0.829 | 0.667 | 0.855 | 0.424 | 0.941 | 4.596 | 0.390 | 0.519 |

Note: AUC, area under the curve; PPV, Positive Predictive Value; NVP, Negative Predictive Value; PLR, Positive Likelihood Ratio; NLR, Negative Likelihood Ratio.

**Table S11. Comparison of performance of different radiologists with and without artificial intelligence assistance**

| **Metrics** | **Expert radiologists (n = 2)** | | |  | **Senior radiologists (n = 4)** | | |  | **Novice radiologists (n = 4)** | | |
| --- | --- | --- | --- | --- | --- | --- | --- | --- | --- | --- | --- |
|  | **Without AI** | **With AI** | **P value** |  | **Without AI** | **With AI** | **P value** |  | **Without AI** | **With AI** | **P value** |
| **Sensitivity (95% Cl)** | 0.690 (0.644-0.737) | 0.881 (0.834-0.928) | ＜0.001 |  | 0.619 (0.581-0.657) | 0.810 (0.771-0.848) | ＜0.001 |  | 0.546 (0.468-0.623) | 0.810 (0.775-0.843) | ＜0.001 |
| **Specificity (95% Cl)** | 0.772 (0.684-0.860) | 0.874 (0.836-0.911) | 0.082 |  | 0.736 (0.691-0.786) | 0.809 (0.796-0.823) | 0.102 |  | 0.722 (0.584-0.861) | 0.780 (0.757-0.803) | 0.312 |
| **Accuracy (95% Cl)** | 0.761 (0.679-0.843) | 0.875 (0.849-0.901) | 0.031 |  | 0.722 (0.686-0.758) | 0.809 (0.795-0.823) | 0.042 |  | 0.696 (0.586-0.806) | 0.784 (0.763-0.805) | 0.011 |
| **PPV (95% CI)** | 0.332 (0.230-0.435) | 0.531 (0.470-0.593) | ＜0.001 |  | 0.278 (0.248-0.309) | 0.405 (0.382-0.428) | ＜0.001 |  | 0.296 (0.192-0.400) | 0.373 (0.346-0.399) | ＜0.001 |
| **NPV (95% CI)** | 0.939 (0.925-0.954) | 0.979 (0.971-0.986) | 0.680 |  | 0.924 (0.920-0.927) | 0.964 (0.957-0.971) | 0.831 |  | 0.905 (0.891-0.918) | 0.962 (0.955-0.969) | 0.793 |
| **F1-Score (95% CI)** | 0.447 (0.343-0.551) | 0.662 (0.627-0.696) | ＜0.001 |  | 0.382 (0.361-0.404) | 0.540 (0.513-0.567) | ＜0.001 |  | 0.358 (0.291-0.426) | 0.510 (0.481-0.539) | ＜0.001 |
| **PLR (95% CI)** | 3.172 (1.746-4.597) | 7.129 (5.383-8.876) | ＜0.001 |  | 2.420 (2.043-2.796) | 4.262 (3.846-4.678) | ＜0.001 |  | 2.765 (1.509-4.020) | 3.729 (3.327-4.131) | ＜0.001 |
| **NLR (95% CI)** | 0.404 (0.298-0.510) | 0.136 (0.088-0.183) | ＜0.001 |  | 0.515 (0.489-0.542) | 0.236 (0.188-0.283) | ＜0.001 |  | 0.642 (0.546-0.738) | 0.245 (0.198-0.291) | ＜0.001 |

Note: AUC, area under the curve; PPV, Positive Predictive Value; NVP, Negative Predictive Value; PLR, Positive Likelihood Ratio; NLR, Negative Likelihood Ratio.

**Table S12. Cox Regression Analysis for OS and DFS Across Training and Validation Cohorts**

| **Cohort** | **OS** | | | **DFS** | | |
| --- | --- | --- | --- | --- | --- | --- |
|  | **HR** | **95%CI** | **P value** | **HR** | **95%CI** | **P value** |
| **Training set** | 0.489 | 0.394 to 0.606 | ＜0.001 | 0.472 | 0.383 to 0.581 | ＜0.001 |
| **Validation set I** | 0.585 | 0.435 to 0.785 | ＜0.001 | 0.520 | 0.393 to 0.686 | ＜0.001 |
| **Validation set II** | 0.447 | 0.313 to 0.638 | ＜0.001 | 0.378 | 0.271 to 0.528 | ＜0.001 |
| **Peritoneal metastasis validation cohort** | 0.476 | 0.331 to 0.685 | ＜0.001 | 0.368 | 0.258 to 0.526 | ＜0.001 |
| **Postoperative peritoneal recurrence validation cohort** | 0.712 | 0.571 to 0.889 | 0.003 | 0.722 | 0.584 to 0.894 | 0.003 |
